# Supplementary material for: Characterization and AAV-mediated CRB gene augmentation in human-derived CRB1KO and CRB1KOCRB2+/− retinal organoids
Source: Mol Ther Methods Clin Dev. 2023 Oct 10;31:101128. doi: 10.1016/j.omtm.2023.101128 (PMC10597801; doi:10.1016/j.omtm.2023.101128)
Supplement: Document S1. Figures S1–S5 and Tables S1–S3 [file mmc1.pdf]

OMTM, Volume *31*

Supplemental information

Characterization and AAV-mediated *CRB* gene augmentation in human-derived *CRB1*^{KO} and *CRB1*^{KO}*CRB2*$^{+/-}$ retinal organoids

Nanda Boon, Xuefei Lu, Charlotte A. Andriessen, Michaela Orlovà, Peter M.J. Quinn, Camiel J.F. Boon, and Jan Wijnholds

Table S1. hiPSC line information, related to Figure S1.

Previously published	Description	Gender
LUMC04iCTRL10	ISO-4.10 = Isogenic control	Male
No	*CRB1*^{KO} CL19 = homozygous c.500del ; p.(Ser44Serfs*)	Male
No	*CRB1*^{KO} CL26 = homozygous c.500del ; p.(Ser44Serfs*)	Male
No	*CRB1*^{KO} CL72 = homozygous c.500del ; p.(Ser44Serfs*)	Male
No	*CRB1*^{KO}*CRB2*$^{+/-}$ CL4 =<br>*CRB1*: homozygous c.500del ; p.(Ser44Serfs*),<br>*CRB2*: heterozygous c.576_598del; p.(Cys193Argfs*)	Male
No	*CRB1*^{KO}*CRB2*$^{+/-}$ CL9 =<br>*CRB1*: homozygous c.500del ; p.(Ser44Serfs*),<br>*CRB2*: heterozygous c.576_598del; p.(Cys193Argfs*)	Male
No	*CRB1*^{KO}*CRB2*$^{+/-}$ CL17 =<br>*CRB1*: homozygous c.498_507delinsTGCC ; p.(Ser44Lysfs*),<br>*CRB2*: heterozygous c.583_584del; p.(His195Trpfs*)	Male

Table S2. PCR primer sequences for confirmation mutations.

**Target**		**PCR primers**
CRB1	FW	GACAATGATTGTTCTTGTTCAGACACAGCC
	REV	CATCCACTTCCAAGTCGCAGTGTC
		**Sanger sequencing primers**
CRB1	FW	GGACAAAGACTGTGACAACATGAAAGACC
	REV	GGACACAGAAGCAGGAGTAACCATC
**Target**		**NGS primers (5' to 3') (Illumina adapter overhang in blue)**
CRB2	FW	GATGTGTATAAGAGACAGGTGTCCATCCTGCACCCTGTG
	REV	CGTGTGCTCTTCCGATCTTCGCTCACCCGTTGACCAGGT

Table S3. Barcode PCR primers used in the NGS amplicon sequence analysis.

**Barcode name**	**Primer Sequence (5' to 3')**	**Barcode in primer**	**Read in Miseq**
719	CAAGCAGAAGACGGCATACGAGAT | TACTACGC | GTGACTGGAGTTCAGACGTGTGCTCTTCCGATCT	TACTACGC	TACTACGC
720	CAAGCAGAAGACGGCATACGAGAT | AGGCTCCG | GTGACTGGAGTTCAGACGTGTGCTCTTCCGATCT	AGGCTCCG	AGGCTCCG
721	CAAGCAGAAGACGGCATACGAGAT | GCAGCGTA | GTGACTGGAGTTCAGACGTGTGCTCTTCCGATCT	GCAGCGTA	GCAGCGTA
722	CAAGCAGAAGACGGCATACGAGAT | GAGCGCTA | GTGACTGGAGTTCAGACGTGTGCTCTTCCGATCT	GAGCGCTA	GAGCGCTA
508	AATGATACGGCGACCACCGAGATCTACAC | GTACTGAC | TCGTCGGCAGCGTCAGATGTGTATAAGAGACAG	GTACTGAC	GTACTGAC

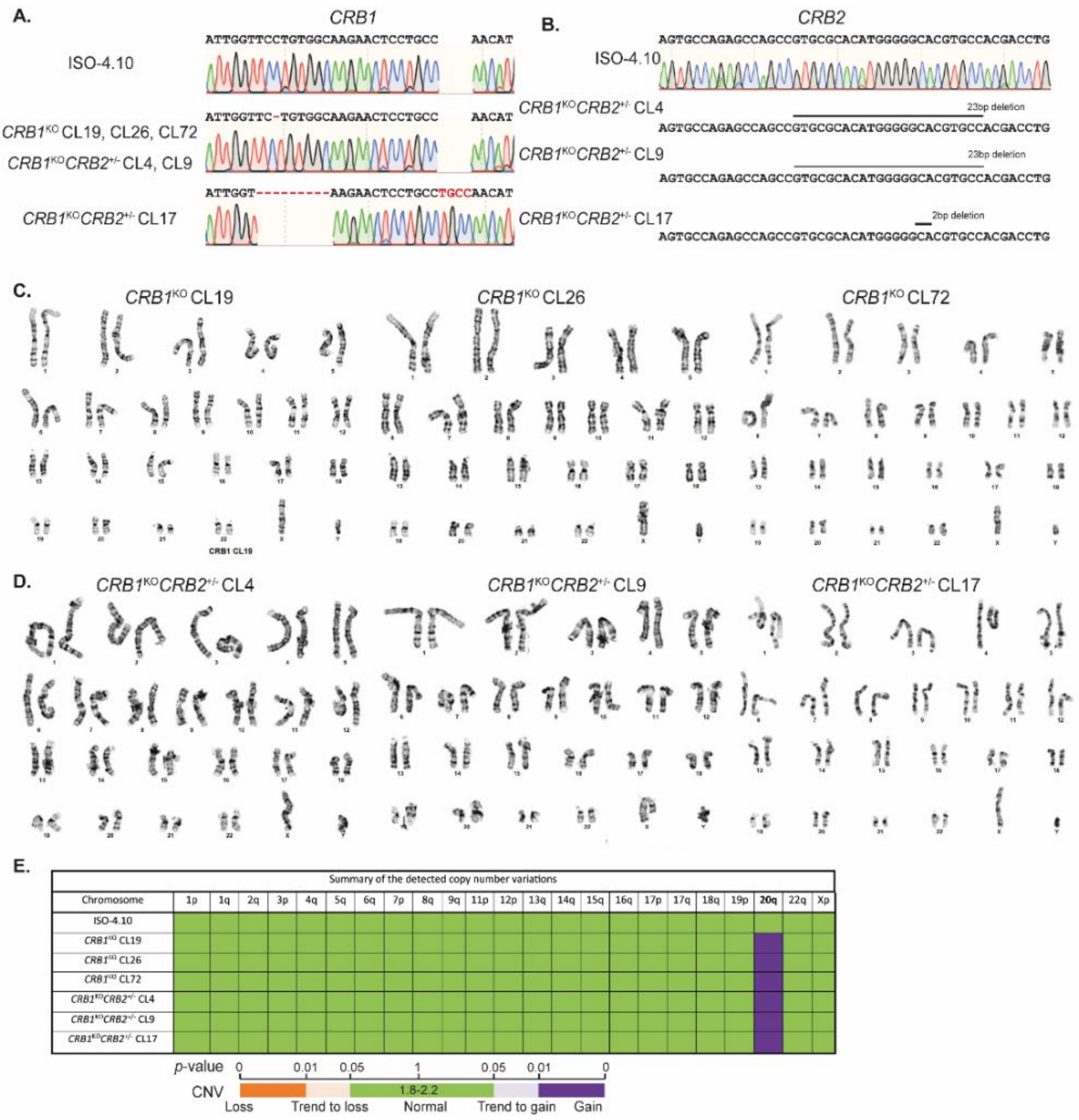

Figure S1: Confirmation of mutations and condition of *CRB1*^{KO} and *CRB1*^{KO}*CRB2*$^{+/-}$ hiPSC. Related to all figures. (A) Confirmation of the homozygous *CRB1* mutation in *CRB1*^{KO} and *CRB1*^{KO}*CRB2*$^{+/-}$ hiPSC using Sanger sequencing, with *CRB1*^{KO}*CRB2*$^{+/-}$ CL17 containing a distinct mutation. (B) Confirmation of the heterozygous *CRB2* mutation in *CRB1*^{KO}*CRB2*$^{+/-}$ hiPSC using Sanger sequencing (upper trace) and an insertion/deletion (INDEL) detection software (ICE) (lower sequences). (C, D) Karyotyping results of (C) *CRB1*^{KO} and (D) *CRB1*^{KO}*CRB2*$^{+/-}$ hiPSC. (E) Meta-analysis of 90% most recurrent abnormalities in hiPSCs showing a gain in copy number variation (CNV) in chromosome 20q for hiPSC used in this study.

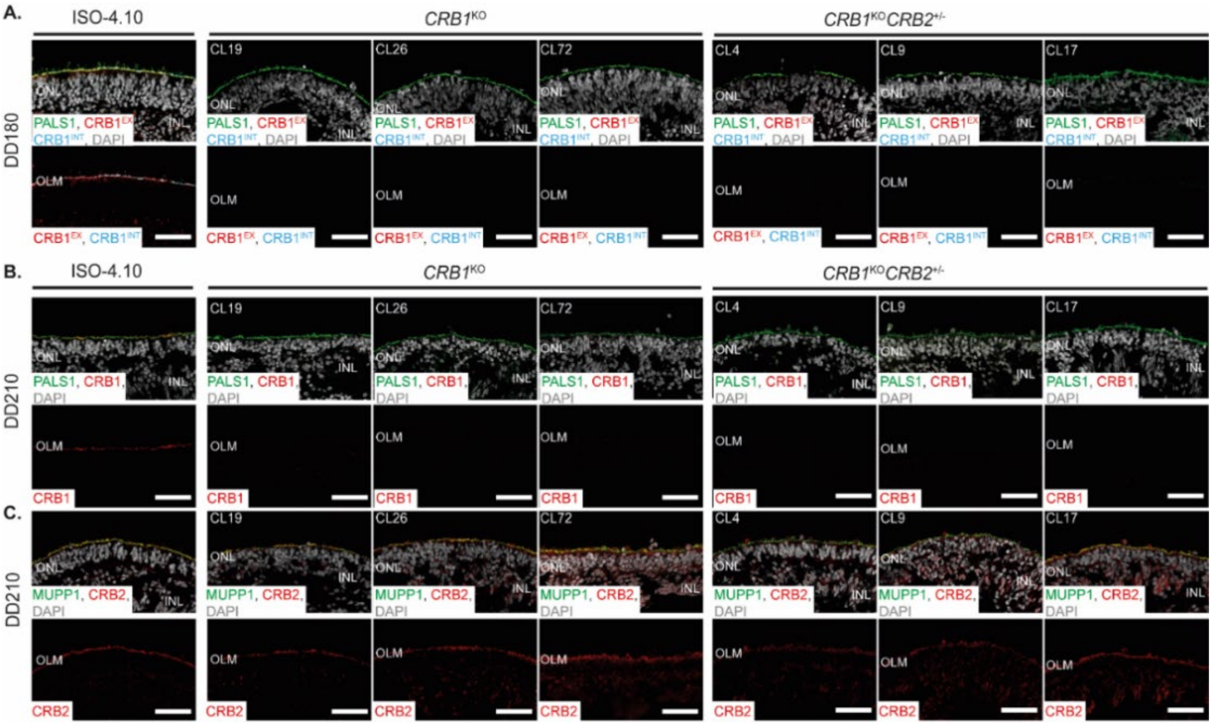

Figure S2: CRB1 is absent in DD180 and DD210 $CRB1^{KO}$ and $CRB1^{KO}CRB2^{+/-}$ retinal organoids. Related to Figure 2. Representative immunohistochemical images of three $CRB1^{KO}$ and three $CRB1^{KO}CRB2^{+/-}$ retinal organoids compared to the isogenic control at DD180 and DD210. (A) DD180 organoids expressing PALS1 (green) in all retinal organoids at the OLM and $CRB1^{EX}$ (red), and $CRB1^{INT}$ (cyan) was only detected in the isogenic control. (B) DD210 retinal organoids stained with PALS1 (green) and CRB1 (red), similar as observed in DD180. (C) MUPP1 (green) and CRB2 (red) is expressed at the OLM in control, $CRB1^{KO}$, and $CRB1^{KO}CRB2^{+/-}$ retinal organoids at DD210. Note: INL = Inner Nuclear Layer, ONL = Outer Nuclear Layer, OLM = Outer Limiting Membrane. Scalebar = 50µm.

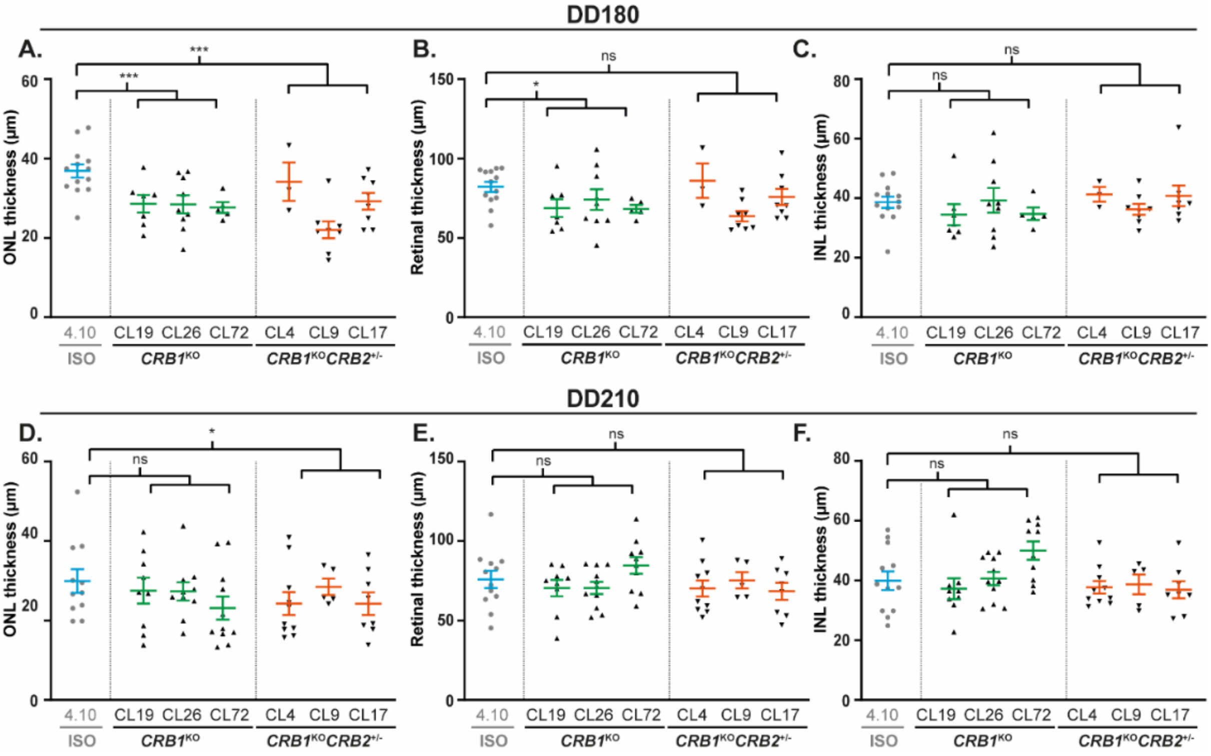

Figure S3: Additional phenotype quantifications of DD180 and DD210 *CRB1*KO and *CRB1*KO*CRB2*+/- retinal organoids. (A, B, C) Quantification of the (A) ONL, (B) retinal, and (C) INL thickness of DD180 retinal organoids. (D, E, F) Quantification of the (D) ONL, (E) retinal, and (F) INL thickness of DD210 retinal organoids. Each datapoint in the graph represents an individual organoid, of which an average has been taken of 3-6 representative images. The standard error of mean (SEM) is derived from these averages. Number of individual organoids used for the quantification per condition at DD180: 4.10 n =14, CRB1^{KO} CL19 n = 7, CL26 n =5, CL72 n = 9, *CRB1*^{KO}*CRB2*$^{+/-}$ CL4 n = 3, CL9 n = 8, CL17 n = 8; and DD210: 4.10 n = 12, CRB1^{KO} CL19 n = 9, CL26 n =10, CL72 n = 11, *CRB1*^{KO}*CRB2*$^{+/-}$ CL4 n = 8, CL17 n = 8 from at least two independent differentiation batches and *CRB1*^{KO}*CRB2*$^{+/-}$ CL9 n = 5 from one differentiation batch. Statical analysis: generalized linear mixed models with $p<0.05$ (*), $p<0.01$ (**), and $p<0.001$ (***).

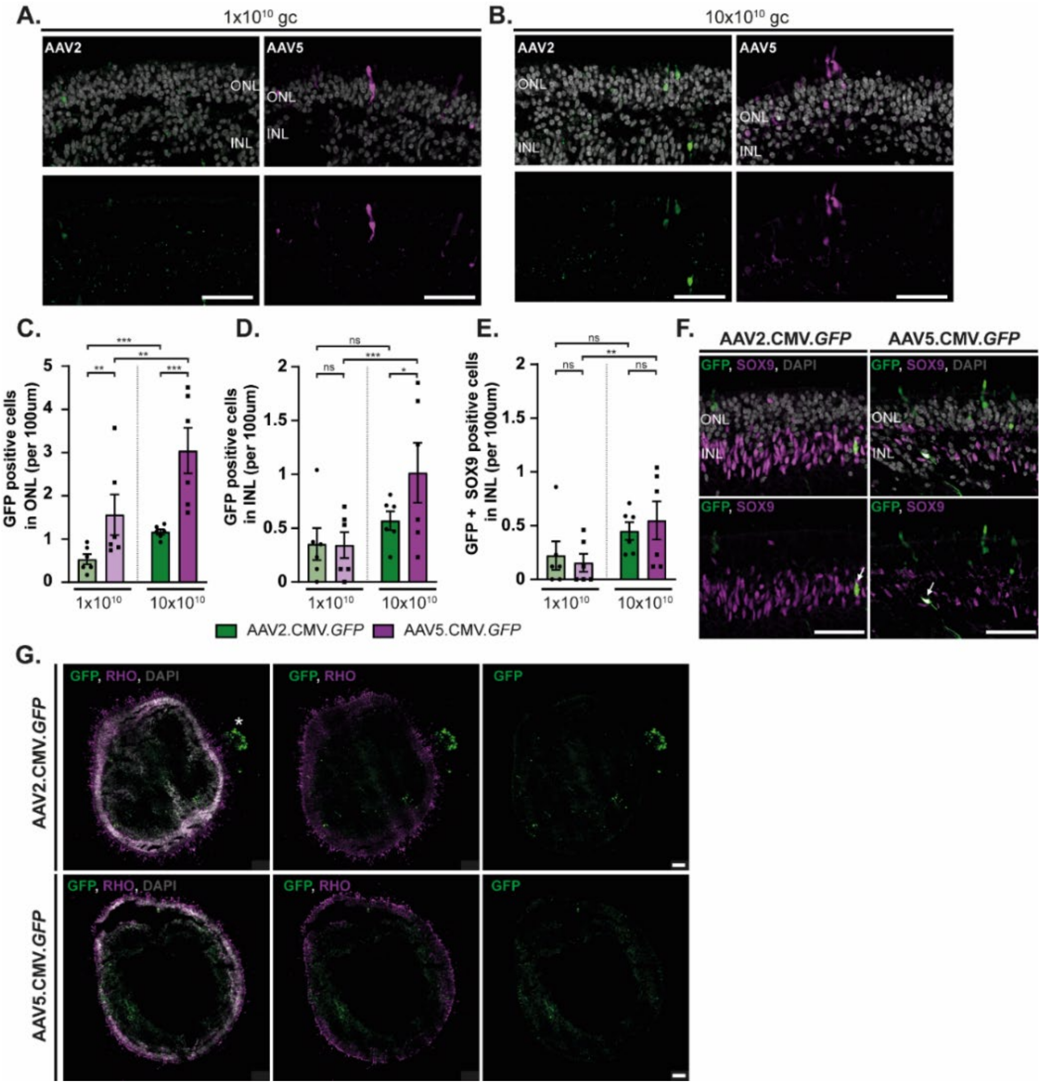

Figure S4: AAV transduction study of control retinal organoids transduced at DD200 with AAV2.CMV.*GFP* or AAV5.CMV.*GFP* (A, B) Representative immunohistochemical images of control retinal organoids transduced with (A) 1x10^{10}gc, or (B) 10x10^{10}gc AAV2.CMV.*GFP* or AAV5.CMV.*GFP*. (C,D, E) Quantification of the number of GFP positive cells in the (D) ONL, (E) INL, and (F) GFP positive and co-localized with SOX9 in the INL (marking Müller glial cells). Statistical analysis was performed within the same AAV capsid (dose-dependent) or within the same AAV titer (comparing AAV2 with AAV5 transduction efficiency). (F, G) Immunohistochemical images of co-localization of AAV.*GFP* with Müller glial cells marker SOX9 (F) and photoreceptor cell marker Rhodopsin (RHO) (G). Arrows indicate colocalization of SOX9 with GFP (F), and the asterisk indicates the RPE (G). Each datapoint in the graph represents individual organoids, of which an average has been taken of 3-6 representative images. The standard error of mean (SEM) is derived from these averages. Number of individual organoids used for quantification per condition for AAV2.CMV.*GFP*: 1x10^{10}gc n = 6, 10x10^{10}gc n = 6; and for AAV5.CMV.*GFP*: 1x10^{10}gc n = 6, 10x10^{10}gc n = 6 from one differentiation batch. Note: INL = Inner Nuclear Layer, ONL = Outer Nuclear Layer. Scalebar = 50µm, statical analysis: generalized linear mixed models with p<0.05 (*), p<0.01 (**), and p<0.001 (***).

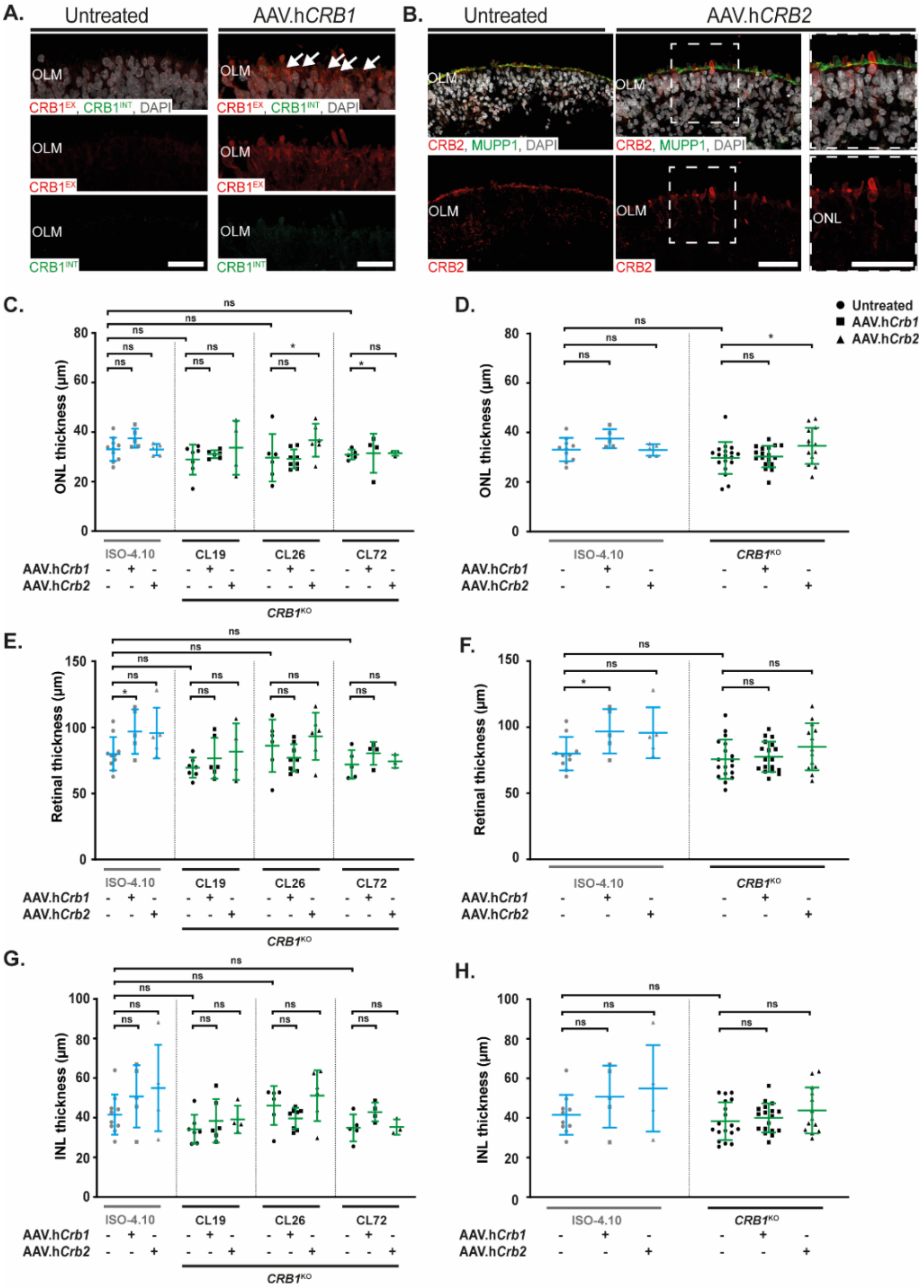

Figure S5: AAV-mediated gene therapy on *CRB1*^{KO} organoids. (A, B) representative immunohistochemical image of (A) CRB1 in untreated and AAV.h*CRB1* and (B) CRB2 in untreated and AAV.h*CRB2* treated *CRB1*^{KO} retinal organoids. (C,D, E, F, G, H) Quantification of the (C, D) ONL thickness, (E, F) the retinal thickness, and (G, H) INL thickness per *CRB1*^{KO} clone (C, E, G) or all *CRB1*^{KO} clones combined (D, F, H). Each datapoint in the graph represents individual organoids, of which an average has been taken of at least three representative images. The standard error of mean (SEM) is derived from these averages. Number of individual organoids used for quantification per condition for untreated: 4.10 n = 10, *CRB1*^{KO} CL19 n = 7, CL26 n = 7, CL72 n = 5; AAV.h*CRB1* treated: 4.10 n = 5, *CRB1*^{KO} CL19 n = 6, CL26 n = 8, CL72 n = 4 from two independent differentiation batches; and AAV.h*CRB2* treated: 4.10 n = 5, *CRB1*^{KO} CL19 n = 4, CL26 n = 6, CL72 n = 3 from one differentiation batch. Note: ONL = Outer Nuclear Layer, OLM = Outer Limiting Membrane. Scalebar = 50µm, statistical analysis: generalized linear mixed models with $p < 0.05$ (*), $p < 0.01$ (**), and $p < 0.001$ (***).
