# Supplementary material for: Characterization and AAV-mediated CRB gene augmentation in human-derived CRB1KO and CRB1KOCRB2+/− retinal organoids
Source: Mol Ther Methods Clin Dev. 2023 Oct 10;31:101128. doi: 10.1016/j.omtm.2023.101128 (PMC10597801; doi:10.1016/j.omtm.2023.101128)
Supplement: Document S2. Article plus supplemental information [file mmc2.pdf]

Molecular Therapy
Methods & Clinical Development
Original Article

Characterization and AAV-mediated *CRB* gene augmentation in human-derived *CRB1*^{KO} and *CRB1*^{KO}*CRB2*$^{+/-}$ retinal organoids

Nanda Boon,[1] Xuefei Lu,[1] Charlotte A. Andriessen,[1] Michaela Orlovà,[1] Peter M.J. Quinn,[1,4] Camiel J.F. Boon,[1,2] and Jan Wijnholds[1,3]

[1]Department of Ophthalmology, Leiden University Medical Center (LUMC), Albinusdreef 2, 2333 ZA Leiden, the Netherlands; [2]Department of Ophthalmology, Amsterdam University Medical Centers, 1000 AE Amsterdam, the Netherlands; [3]Netherlands Institute for Neuroscience, an Institute of the Royal Netherlands Academy of Arts and Sciences (KNAW), Meibergdreef 47, 1105 BA Amsterdam, the Netherlands

The majority of patients with mutations in *CRB1* develop either early-onset retinitis pigmentosa as young children or Leber congenital amaurosis as newborns. The cause for the phenotypic variability in *CRB1*-associated retinopathies is unknown, but might be linked to differences in CRB1 and CRB2 protein levels in Müller glial cells and photoreceptor cells. Here, *CRB1*^{KO} and *CRB1*^{KO}*CRB2*$^{+/-}$ differentiation day 210 retinal organoids showed a significant decrease in the number of photoreceptor nuclei in a row and a significant increase in the number of photoreceptor cell nuclei above the outer limiting membrane. This phenotype with outer retinal abnormalities is similar to *CRB1* patient-derived retinal organoids and *Crb1* or *Crb2* mutant mouse retinal disease models. The *CRB1*^{KO} and *CRB1*^{KO}*CRB2*$^{+/-}$ retinal organoids develop an additional inner retinal phenotype due to the complete loss of CRB1 from Müller glial cells, suggesting an essential role for CRB1 in proper localization of neuronal cell types. Adeno-associated viral (AAV) transduction was explored at early and late stages of organoid development. Moreover, AAV-mediated gene augmentation therapy with AAV.h*CRB2* improved the outer retinal phenotype in *CRB1*^{KO} retinal organoids. Altogether, these data provide essential information for future gene therapy approaches for patients with *CRB1*-associated retinal dystrophies.

INTRODUCTION

Crumbs homologue 1 (CRB1) is a large transmembrane protein initially discovered at the apical membrane of *Drosophila* epithelial cells.[1] The human *CRB1* gene is mapped to chromosome 1q31.3, has 12 identified mRNA transcripts, over 210 kb genomic DNA, and three CRB family members.[2,3] Canonical human CRB1 consists of multiple epidermal growth factor (EGF) and laminin-globular like domains in its large extracellular domain. The short intracellular domain contains an FERM domain juxtaposed to the single transmembrane domain and at the carboxyl-terminus a conserved glutamic acid-arginine-leucine-isoleucine (ERLI) PDZ binding motif.[3–5] A short alternative transcript of *CRB1*, *CRB1-B*, was described encoding a protein with significant extracellular domain overlap with canonical CRB1 while lacking the transmembrane, C-terminal intracellular domain, and a large part of the N-terminal domain.[6] The function of *CRB1-B* in the human retina is not known. In mammals, the CRB family members are CRB1, CRB2, and CRB3A. CRB2 displays a similar protein structure to CRB1, except for a depletion of four EGF domains. The canonical CRB complex is formed by interaction with protein associated with Lin Seven 1 (PALS1), which binds to the conserved carboxy-terminal PDZ domain of CRB.[7,8] The CRB complex is evolutionarily conserved and is important for regulating apical-basal polarity and maintaining cell adhesion.[9]

Inherited retinal dystrophies such as retinitis pigmentosa (RP) or Leber congenital amaurosis (LCA) can be caused by mutations in the *CRB1* gene. Approximately 7%–17% of LCA and 3%–9% of RP patients are reported with mutations in *CRB1*.[10–12] RP is a clinically and genetically heterogeneous disease where children or aged patients experience night blindness that progresses to complete loss of vision,[13] while LCA causes visual impairment in newborns.[14,15] There are over 200 different mutations along the *CRB1* gene described to be causing early-onset RP in children or LCA without a clear genotype-phenotype correlation.[16–21] No treatment possibilities are available for patients with a mutation in the *CRB1* gene. Multiple animal-derived models have been described that mimic the phenotype of *CRB1* patients.[4,22] However, recent immunoelectron microscopy studies have shown that the subcellular localization of CRB1 and CRB2 is different between rodents and humans. In mice, CRB1 is located at the subapical region just above the outer limiting membrane (OLM) of Müller glial cells (MGC) while CRB2 is located at the subapical region of Müller glial and photoreceptor cells.[23] In adult

Received 25 July 2023; accepted 4 October 2023;
https://doi.org/10.1016/j.omtm.2023.101128.

[4]Present address: Edward S. Harkness Eye Institute, Department of Ophthalmology, Columbia University Irving Medical Center/New York-Presbyterian Hospital, New York, NY 10032, USA

Correspondence: Jan Wijnholds, Department of Ophthalmology, Leiden University Medical Center (LUMC), Albinusdreef 2, 2333 ZA Leiden, the Netherlands.
E-mail: j.wijnholds@lumc.nl

This is an open access article under the CC BY license (http://creativecommons.org/licenses/by/4.0/).

non-human-primate retina, human-derived retinal organoids and human fetal retina, both CRB1 and CRB2 are located at the subapical region of Müller glial and photoreceptor cells.[24] In addition, a reappraisal of the phenotype-genotype correlation of 50 patients with regard to canonical CRB1 and the photoreceptor-specific CRB1-B has shown that the retinal phenotype is mainly driven by canonical *CRB1* isoform impairment.[17] These data indicate thus the importance of using human-derived models to study the retinal dystrophy caused by mutations in *CRB1*.

The use of human-induced pluripotent stem cell (hiPSC)-derived models for research is an emerging strategy to explore patient organoid or cell phenotypes *in vitro*. This is specifically of interest for patients with mutations in *CRB1*, because the subcellular localization of the protein is different between rodents and humans. hiPSC can be differentiated into well-defined retinal organoids that recapitulate the development of the fetal retina.[25] Previously, we have shown that *CRB1*-patient-derived retinal organoids frequently show ectopic photoreceptor cells above the OLM and detected less variant CRB1 protein at the OLM of patient-derived retinal organoids at differentiation day 180 (DD180) and DD210.[25–27] In a large clinical cohort, it was shown previously that there is no clear genotype-phenotype correlation for patients with a mutation in the *CRB1* gene and the mutations are distributed along the *CRB1* gene.[20] Here, we describe the use of hiPSC-derived $CRB1^{KO}$ retinal organoids as a candidate model for LCA, where a single nucleotide was deleted by CRISPR-Cas9 in exon 2 resulting in a frameshift with a premature stop codon and thus a knockout of the gene of interest. In addition, $CRB1^{KO}CRB2^{+/-}$ hiPSCs were used, since previous research has shown that concomitant decreased levels of *CRB2* can exacerbate the phenotype in *CRB1* mutant mice.[22,24,28–31] Homozygous mutations were introduced by CRISPR-Cas9 in exon 2 of *CRB1* and a heterozygous mutation in exon 3 of *CRB2*, generating $CRB1^{KO}CRB2^{+/-}$ hiPSC. *CRB* retinal organoids completely lacking CRB1 protein showed an outer and inner retina phenotype mimicking a mild form of LCA. In previous studies, we analyzed the therapeutic efficacy of adeno-associated viral vector (AAV)-*CRB1* and AAV-*CRB2* in RP patient *CRB1* retinal organoids that expressed 4-fold reduced levels of variant CRB1 protein.[26,27] AAV-*CRB2* and AAV-*CRB1* transduction onto these *CRB1* patient retinal organoids prevents morphological and transcriptional aberrations.[26,27] Here, the candidate LCA $CRB1^{KO}$ retinal organoids that lack expression of CRB1 protein were used for AAV-mediated h*CRB1* or h*CRB2* gene augmentation therapy.

RESULTS

Generation of $CRB1^{KO}$ and $CRB1^{KO}CRB2^{+/-}$ hiPSC from the isogenic control

From the control iPSC line LUMC4iCTRL10 (now called: ISO-4.10), three $CRB1^{KO}$ and three $CRB1^{KO}CRB2^{+/-}$ clones were generated using CRISPR-Cas9 technology (Applied StemCell). In short, guide RNAs were designed to target exon 2 of *CRB1* and exon 3 of *CRB2*. For the $CRB1^{KO}$, three independent homozygous subclones (CL19, CL26, and CL72) carried a homozygous deletion (c.500del), resulting in a frameshift with premature stop p.(Ser44Serfs*) for the *CRB1* gene (Figure S1A). For $CRB1^{KO}CRB2^{+/-}$, two clones (CL4 and CL9) carried the same homozygous c.500del mutation and the CL17 iPSC clone carried a homozygous c.498_507delinsTGCC mutation in the *CRB1* gene, both mutations result in a frameshift with premature translation stop of CRB1 (Figure S1A). For the *CRB2* gene, the $CRB1^{KO}CRB2^{+/-}$ clones showed heterozygous mutations targeting exon 3 (Figure S1B). Sanger sequencing results of the *CRB2* gene were imported in the Inference of CRISPR Edits (ICE) analysis tool to analyze the CRISPR edits with NGS-quality (ICE v3, Synthego). $CRB1^{KO}CRB2^{+/-}$ clones 4 and 9 show a heterozygous 23-bp deletion (c.576_598del; p.(Cys193Argfs*)) in exon 3 of the *CRB2* gene (Figure S1B), resulting in a frameshift with an alternative translation of CRB2. $CRB1^{KO}CRB2^{+/-}$ clone 17 shows a heterozygous 2-bp deletion (c.583_584del; p.(His195Trpfs*)) in exon 3 of the *CRB2* gene (Figure S1B), resulting in a frameshift with an alternative translation of CRB2 and the normal protein translation. Karyotyping of all clones showed a normal karyotype (Figures S1C and S1D), but the $CRB1^{KO}$ and $CRB1^{KO}CRB2^{+/-}$ showed a commonly observed gain in copy number variation (CNV) on chromosome 20q (Figure S1E). The biological significance of such recurrent abnormalities is still discussed,[32] and further research is required to define this. These *CRB* and isogenic (ISO-4.10) hiPSC lines were differentiated into $CRB1^{KO}$, $CRB1^{KO}CRB2^{+/-}$, and isogenic control retinal organoids.

$CRB1^{KO}$ and $CRB1^{KO}CRB2^{+/-}$ retinal organoids show an inner retinal phenotype at DD210

The $CRB1^{KO}$, $CRB1^{KO}CRB2^{+/-}$, and isogenic control iPSC lines were differentiated into defined retinal organoids with outer segment-like structures up to, at least, DD210 based on bright field images of the retinal organoids in culture (Figures 1A–1C'). In addition, a laminated retina with a clear outer nuclear layer (ONL) marked by rhodopsin-positive photoreceptor cells (Figure 1D) and an inner nuclear layer (INL) indicated with SOX9-positive Müller glial cells and ISLET1-2 positive rod and ON-cone bipolar cells (Figure 1E) were observed in all retinal organoids at DD210. In recent single-cell RNA sequencing (scRNA-seq) studies we observed variable numbers of rod and cone photoreceptors in retinal organoids cultured under similar conditions.[26] However, we observed a less defined alignment of SOX9 and ISLET1-2 positive cells in the inner retina of $CRB1^{KO}$ and $CRB1^{KO}CRB2^{+/-}$ compared with isogenic control retinal organoids, possibly due to the complete loss of canonical CRB1 in Müller glial cells (and photoreceptors). This was previously not observed in *CRB1* patient-derived retinal organoids that expressed strongly decreased but existing basic levels of variant CRB1.[25–27] The data on the $CRB1^{KO}$ and $CRB1^{KO}CRB2^{+/-}$ retinal organoids at DD210 suggests the disrupted localization of rod and ON-cone bipolar cells. In summary, whereas in previous studies on *CRB1* patient-derived retinal organoids with missense mutations we observed disruptions of the photoreceptor layer, the $CRB1^{KO}$ and $CRB1^{KO}CRB2^{+/-}$ retinal organoids show at DD210 an extended retinal phenotype that includes disruptions of the inner retina.

$CRB1^{KO}$ and $CRB1^{KO}CRB2^{+/-}$ retinal organoids show an outer retinal phenotype at DD180 and DD210

To verify that the $CRB1^{KO}$ mutation caused a complete loss of CRB1 protein expression, immunohistochemical staining of CRB1 on

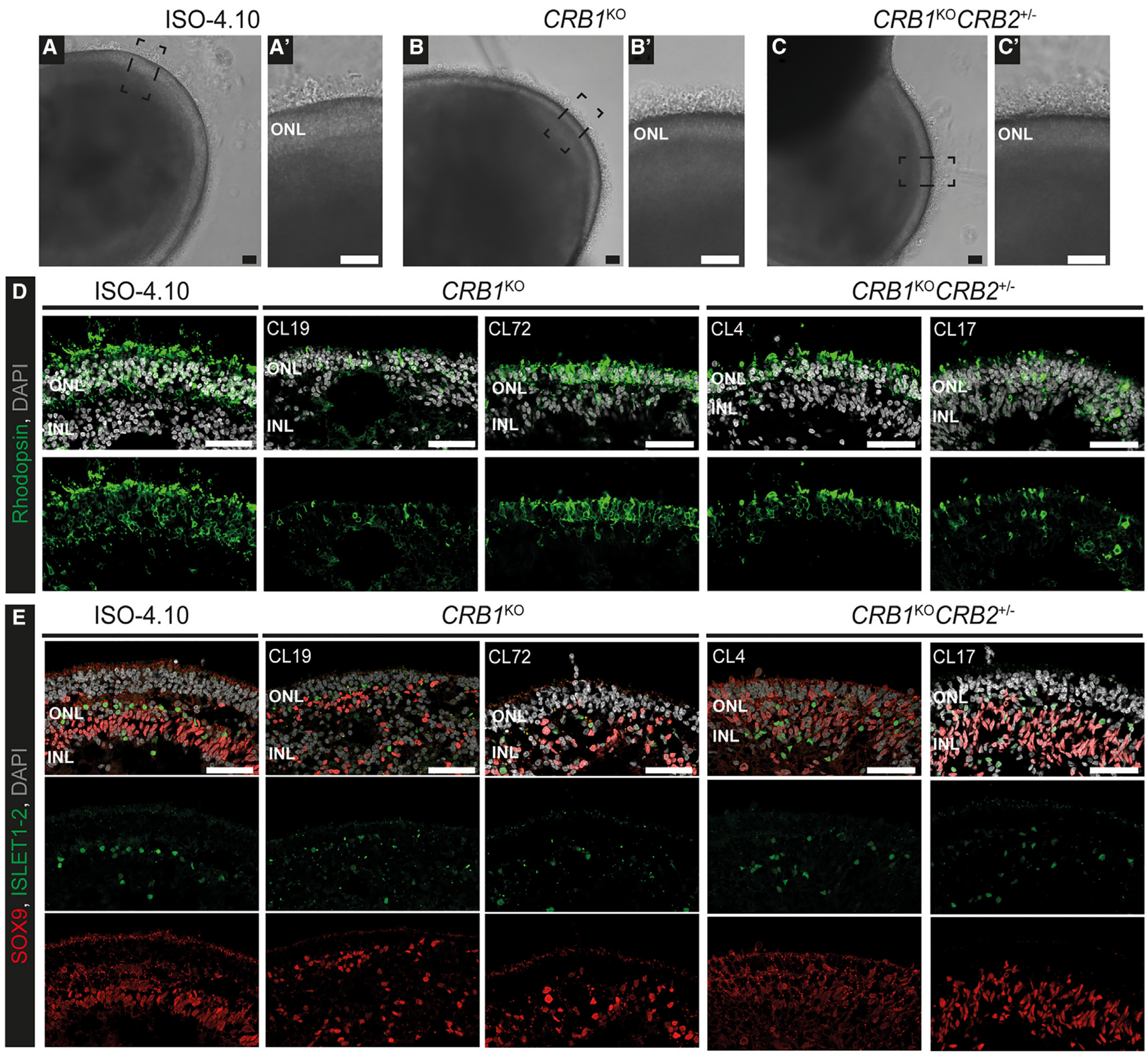

Figure 1. $CRB1^{KO}$ and $CRB1^{KO}CRB2^{+/-}$ retinal organoids at DD210

(A–C) Representative brightfield images of the (A) isogenic control and (B) $CRB1^{KO}$ and (C) $CRB1^{KO}CRB2^{+/-}$ retinal organoids at DD210, with a zoom-in of the outer segment-like structures in the boxed areas (A′, B′, C′). (D and E) Representative immunohistochemistry images of DD210 isogenic control and two $CRB1^{KO}$ and $CRB1^{KO}CRB2^{+/-}$ retinal organoids stained with (D) rhodopsin and (E) SOX9 (red) and ISLET1-2 (green). INL, inner nuclear layer; ONL, outer nuclear layer. Scale bar, 50 μm.

DD180 and DD210 isogenic control, $CRB1^{KO}$, and $CRB1^{KO}CRB2^{+/-}$ retinal organoids was performed. CRB1 and MUPP1 staining at the OLM was observed in the isogenic control at DD180 (Figure 2A). No CRB1 staining was detected in $CRB1^{KO}$ and $CRB1^{KO}CRB2^{+/-}$ retinal organoids, whereas MUPP1 was detected at the OLM (Figure 2A). Both extracellular and intracellular CRB1 antibodies detected no CRB1 staining at the OLM of $CRB1^{KO}$ and $CRB1^{KO}CRB2^{+/-}$ DD180 retinal organoids (Figure S2A). Also, at DD210, no CRB1 was detected in $CRB1^{KO}$ and $CRB1^{KO}CRB2^{+/-}$ retinal organoids, whereas PALS1 was detected at the OLM (Figure S2B). Moreover, CRB2 protein was detected at the OLM in DD180 and DD210 isogenic control, $CRB1^{KO}$, and $CRB1^{KO}CRB2^{+/-}$ retinal organoids (Figures 2B and S2C). Localization of CRB2 was also detected at the OLM of $CRB1^{KO}CRB2^{+/-}$ retinal organoids that harbor one wild-type and one knockout allele of *CRB2*.

When analyzing the $CRB1^{KO}$ and $CRB1^{KO}CRB2^{+/-}$ retinal organoids in more detail, a significantly increased number of photoreceptor nuclei above the OLM was observed compared with the isogenic control at

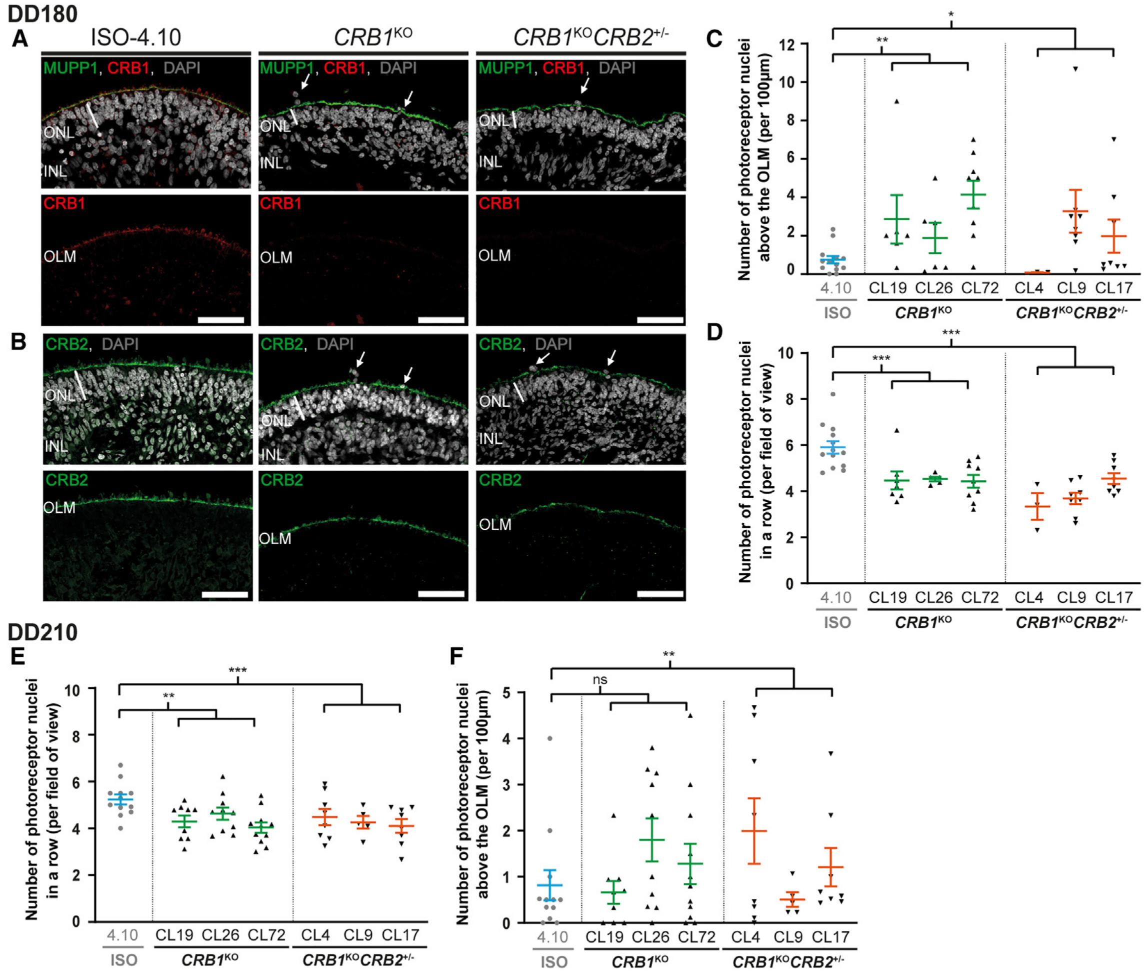

Figure 2. *CRB1*^{KO} and *CRB1*^{KO}*CRB2*$^{+/-}$ retinal organoids show more photoreceptor nuclei above the outer limiting membrane compared with the isogenic control at DD180 and DD210

Representative immunohistochemical images of (A) CRB1 (red) co-stained with MUPP1 (green) and (B) CRB2 (green) at the OLM of DD180 control, *CRB1*^{KO}, and *CRB1*^{KO}*CRB2*$^{+/-}$ retinal organoids. (C) Quantification of the number of photoreceptor nuclei above the OLM and (D) number of photoreceptor nuclei in a row of DD180 control, *CRB1*^{KO}, and *CRB1*^{KO}*CRB2*$^{+/-}$ retinal organoids. (E) Quantification of the number of photoreceptor nuclei above the OLM and (F) number of photoreceptor nuclei in a row of DD210 control, *CRB1*^{KO}, and *CRB1*^{KO}*CRB2*$^{+/-}$ retinal organoids. Each datapoint in the graph represents individual organoids, of which an average has been taken of at least three representative images. The standard error of the mean (SEM) is derived from these averages. Number of individual organoids used for the quantification per condition at DD180: 4.10 n = 14, *CRB1*^{KO} CL19 n = 7, CL26 n = 5, CL72 n = 9, *CRB1*^{KO}*CRB2*$^{+/-}$ CL4 n = 3, CL9 n = 8, CL17 n = 8; and DD210: 4.10 n = 12, *CRB1*^{KO} CL19 n = 9, CL26 n = 10, CL72 n = 11, *CRB1*^{KO}*CRB2*$^{+/-}$ CL4 n = 8, CL17 n = 8 from at least two independent differentiation batches and *CRB1*^{KO}*CRB2*$^{+/-}$ CL9 n = 5 from one differentiation batch. INL, inner nuclear layer; ONL, outer nuclear layer; OLM, outer limiting membrane. Scale bar, 50 μm, statistical analysis: generalized linear mixed models with $p < 0.05$ (*), $p < 0.01$ (**), and $p < 0.001$ (***).

DD180 (Figure 2C). Moreover, a statistically significant decrease in the number of photoreceptor nuclei in a row and ONL thickness was observed in DD180 *CRB1*^{KO} and *CRB1*^{KO}*CRB2*$^{+/-}$ retinal organoids compared with the isogenic control (Figures 2D and S3A). No difference was observed for the INL thickness nor the retinal thickness in *CRB1*^{KO} and *CRB1*^{KO}*CRB2*$^{+/-}$ retinal organoids (Figures S3B and S3C). At DD210, the number of photoreceptor nuclei in a row was still significantly decreased in *CRB1*^{KO} and *CRB1*^{KO}*CRB2*$^{+/-}$ retinal organoids (Figure 2E). However, there were fewer photoreceptor nuclei above the OLM in *CRB1*^{KO} and *CRB1*^{KO}*CRB2*$^{+/-}$ retinal organoids, though still statistically significant for *CRB1*^{KO}*CRB2*$^{+/-}$ retinal organoids compared with the isogenic control (Figure 2F). Again, no statistically significant difference in INL thickness and retinal thickness was observed at DD210 (Figures S3E and S3F). The data indicate that an outer retinal phenotype was observed at DD180 and DD210 retinal organoids, which is comparable to previously observed outer retinal phenotype in *CRB1* patient-derived retinal organoids and in *Crb1* mutant mouse models. Together these data define outcome measures for assessing therapeutic efficacy in *CRB1*^{KO} and *CRB1*^{KO}*CRB2*$^{+/-}$ retinal organoids.

AAV5.CMV.*GFP* and AAV2.CMV.*GFP* transduce control retinal organoids more efficiently at DD135 than at DD200

Previously, we have shown that serotype AAV2/5.CMV.*GFP* (with inverted terminal repeats from AAV2 and capsid from AAV5, from now: AAV5.CMV.*GFP*) was more efficient than AAV2/2.CMV.*GFP* (with inverted terminal repeats from AAV2 and capsid from AAV2, from now: AAV2.CMV.*GFP*) in transducing Müller glial cells at DD120.[26] Here, the tropism of these two serotypes was investigated in control retinal organoids transduced at later timepoints: DD135 or DD200.

At DD135, control retinal organoids were transduced with 1×10^{10} genome copies (gc), 6.6×10^{10} gc, or 10×10^{10} gc of AAV2.-CMV.*GFP* or AAV5.CMV.*GFP* and analyzed using immunohistochemistry after 3 weeks in culture. A dose-dependent increase of GFP-positive cells was observed when control organoids were treated with AAV5.CMV.*GFP* or AAV2.CMV.*GFP* at DD135 (Figures 3A–3C). The AAV-treated retinal organoids were quantified for the number of GFP-positive cells in the ONL and INL. AAV2.CMV.*GFP* significantly transduced more cells in the ONL at the two highest titers (Figure 3D), whereas AAV5.CMV.*GFP* transduced significantly more cells in the INL at 6.6×10^{10}gc (Figure 3E). Co-staining with photoreceptor marker (OTX2) and Müller glial cells markers (CRALBP) confirmed the transduction of both cell types in AAV2.CMV.*GFP* as well as AAV5.CMV.*GFP* transduced organoids (Figures 3F and 3G). These data are in accordance with what was observed when control retinal organoids were transduced at DD120.

Next, we investigated whether transduction with the same AAV capsids at a later time point would influence the tropism. Here, control retinal organoids were transduced with 1×10^{10}gc or 10×10^{10}gc at DD200 with AAV2.CMV.*GFP* or AAV5.CMV.*GFP*. Again, a dose-dependent increase in GFP-positive cells transduced in the ONL was observed for AAV2.CMV.*GFP* and AAV5.-CMV.*GFP*-treated retinal organoids (Figures 4A and 4B). This dose-dependent increase was statistically significant for both capsids in the ONL (Figure S4C) and only for AAV5.CMV.*GFP* in the INL (Figure S4D). Moreover, AAV5.CMV.*GFP* infected significantly more cells in the ONL than AAV2.CMV.*GFP* at both titers (Figure S4C). A small but statistically significant increase in GFP-positive cells in the INL was observed at the dose of 10×10^{10}gc AAV5.CMV.*GFP* (Figure S4D).

However, the number of GFP-positive cells colocalizing with SOX9-marked Müller glial cells showed no significant increase at the dose of 10×10^{10}gc AAV5.CMV.*GFP* (Figure S4E). Both AAV serotypes showed colocalization with SOX9-positive Müller glial cells (Figure S4F). Interestingly, within relatively large retinal organoids only a few cells were transduced with AAV2.CMV.*GFP* or with AAV5.CMV.*GFP* at DD200 (Figure S4G), especially when compared with previous efficient transductions at DD135 and DD120.[26] In previous studies, we transduced RP patient *CRB1* retinal organoids at DD120.[26] We therefore transduced the candidate LCA *CRB1*^{KO} retinal organoids as well at DD120.

AAV-mediated *CRB2* gene augmentation therapy improves the outer retinal phenotype in *CRB1*^{KO} retinal organoids

For gene therapy purposes, *CRB1*^{KO} retinal organoids were treated at DD120 with 3.3×10^{10} gc AAV5.CMVmin.h*CRB1* or AAV5. CMV.h*CRB2* (from now: AAV.h*CRB1* and AAV.h*CRB2*, respectively) and subsequently analyzed at DD210 using immunohistochemistry. First, the isogenic control was treated with AAV.h*CRB* to determine whether there is a positive or negative effect of the gene therapy on control retinal organoids. No difference in retinal lamination or localization of MUPP1 at the OLM was observed after AAV.h*CRB* treatment of the isogenic control (Figures 4A, 4B, and 4C). Also no statistically significant difference was observed after AAV.h*CRB1* nor with AAV.h*CRB2* treatment of the isogenic control for the number of photoreceptor nuclei in a row and the photoreceptor nuclei above the OLM (Figures 4D and 4E).

Next, AAV-mediated gene augmentation therapy was performed on three *CRB1*^{KO} iPSC lines differentiated into retinal organoids. AAV transduction was performed on two independent differentiation batches, one batch with and one without additional co-infection of 3.3×10^{10} gc AAV5.CMV.*GFP*. The results of both experiments were pooled. Immunohistochemical analysis at DD210 showed proper lamination and MUPP1 localization at the OLM after AAV.h*CRB* treatment (Figures 4A, 4B, and 4C). AAV.h*CRB1* and AAV.h*CRB2* expression has been confirmed using immunohistochemistry at the OLM after AAV.*hCRB* treatment in *CRB1*^{KO} (Figures S5A and S5B) and in *CRB1* patient-derived retinal organoids.[26] In previous studies on *Crb2* conditional knockout retina, when targeting only photoreceptors, human recombinant CRB2 protein expression was mainly found in the inner segments of photoreceptors, whereas targeting photoreceptors and Müller glial cells, recombinant CRB2 protein expressed mainly at the OLM.[33] A statistically significant increase in the number of photoreceptor nuclei in a row after AAV.h*CRB2* treatment was observed for *CRB1*^{KO} CL19 and CL26 compared with the untreated *CRB1*^{KO} (Figure 4D). This improvement was more pronounced when the three *CRB1*^{KO} clones were combined (Figure 4E). In addition, a small, but not statistically significant, decrease in number of photoreceptor nuclei above the OLM was observed after AAV.h*CRB2* treatment of the combined *CRB1*^{KO} retinal organoids (Figure 4G). In previous studies, AAV-*CRB2*, but not AAV-*CRB1*, gene augmentation in *CRB1* patient retinal organoids transduced at DD120 and analyzed at DD210 showed statistical decreased numbers of photoreceptor nuclei outside the OLM.[26] No statistically significant improvement was observed after AAV.h*CRB1* treatment of *CRB1*^{KO} retinal organoids (Figures 4D–4G and S5C–S5H). For the ONL thickness, retinal thickness, and INL thickness no statistically significant differences were observed with AAV.h*CRB* treatment of *CRB1*^{KO} retinal organoids (Figures S5C–S5H). Altogether, the data show an improvement of the outer retinal phenotype of *CRB1*^{KO} retinal organoids after treatment with AAV.h*CRB2* in DD210 retinal organoids.

DISCUSSION

In this study, we have shown that the complete loss of CRB1 in human *CRB1*^{KO} and *CRB1*^{KO}*CRB2*$^{+/-}$ retinal organoids results in

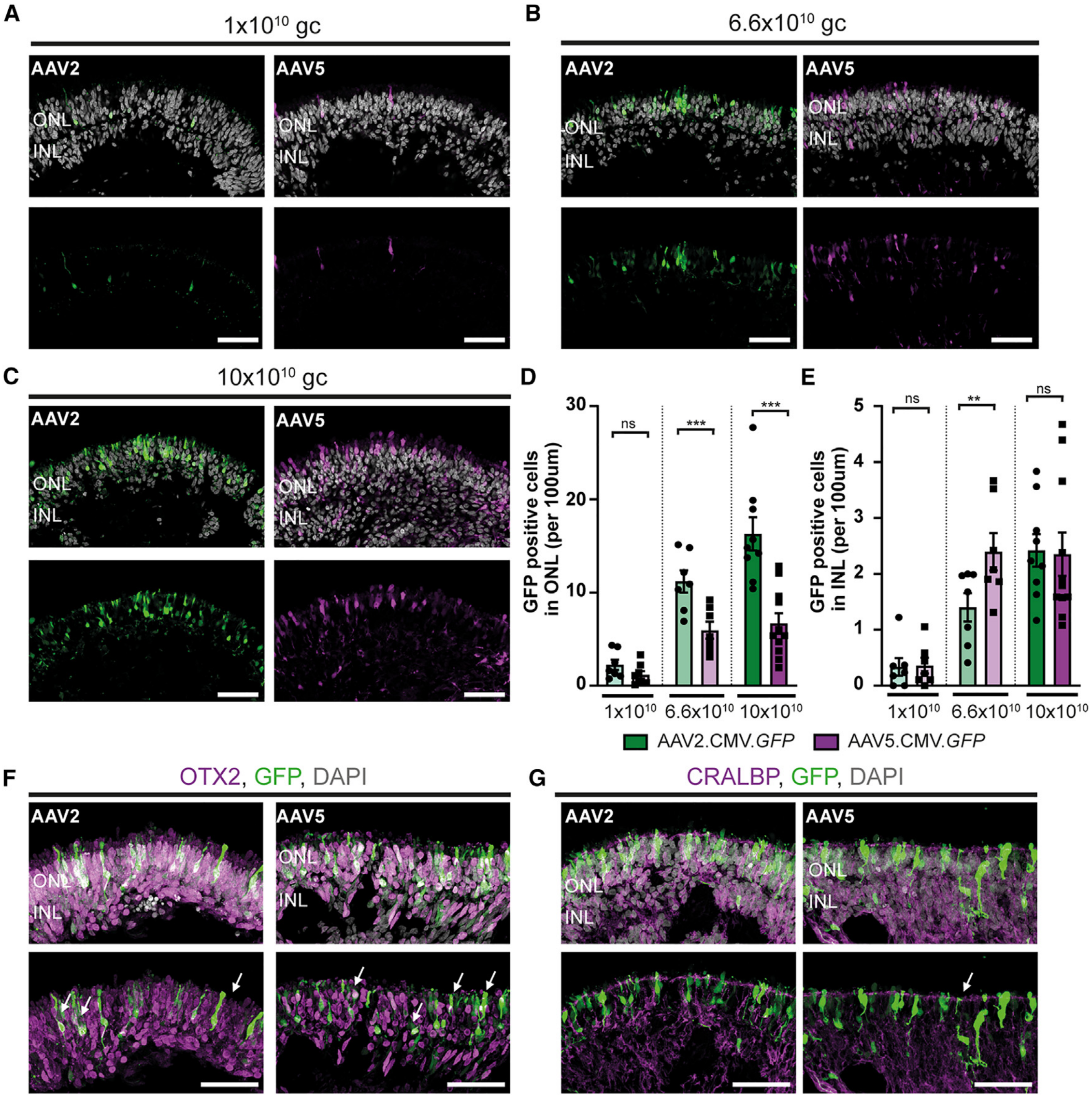

Figure 3. AAV transduction study of control retinal organoids transduced at DD135 with AAV2.CMV.*GFP* or AAV5.CMV.*GFP*

(A–C) Representative immunohistochemical images of control retinal organoids transduced with (A) 1×10^{10} gc, (B) 6.6×10^{10} gc, or (C) 10×10^{10} gc AAV2.CMV.*GFP* or AAV5.CMV.*GFP*. (D and E) Quantification of the number of GFP-positive cells in the (D) ONL and (E) INL. (F and G) Immunohistochemical images of colocalization of AAV.*GFP* with photoreceptor marker OTX2 (F) or Müller glial cell marker CRALBP (G). Each datapoint in the graph represents individual organoids, of which an average has been taken of 3–6 representative images. The standard error of the mean (SEM) is derived from these averages. Number of individual organoids used for quantification per condition for AAV2.CMV.*GFP*: 1×10^{10} gc n = 7, 6.6×10^{10} gc n = 7, 10×10^{10} gc n = 10; and for AAV5.CMV.*GFP*: 1×10^{10} gc n = 9, 6.6×10^{10}gc n = 7, 10×10^{10}gc n = 11 from at least two independent differentiations. INL, inner nuclear layer; ONL, outer nuclear layer. Scale bar, 50 μm, statical analysis: generalized linear mixed models with $p < 0.05$ (*), $p < 0.01$ (**), and $p < 0.001$ (***).

degeneration of the inner and outer retina, whereas *CRB1* RP patient-derived retinal organoids carrying a missense mutation showed strongly reduced levels of variant CRB1 and degeneration of the outer retina.[25–27] We also show that AAV transduction efficiency of retinal organoids depends on the time point of retinal organoid development. In addition, an improved outer retina phenotype of *CRB1*^{KO} retinal organoids was observed after AAV.h*CRB2* transduction.

Here, in order to generate a model for a mild form of LCA, CRISPR-Cas9 was used to generate *CRB1*^{KO} iPSC with a nucleotide deletion in exon 2 of the *CRB1* gene. The nucleotide deletion caused a frameshift resulting in a premature protein translation stop codon. Immunohistochemical analysis confirmed the complete loss of CRB1 in *CRB1*^{KO} retinal organoids at DD180 and DD210. A decreased number of photoreceptor nuclei in a row in the ONL and an increased number of photoreceptor cell nuclei above the OLM were observed at DD180

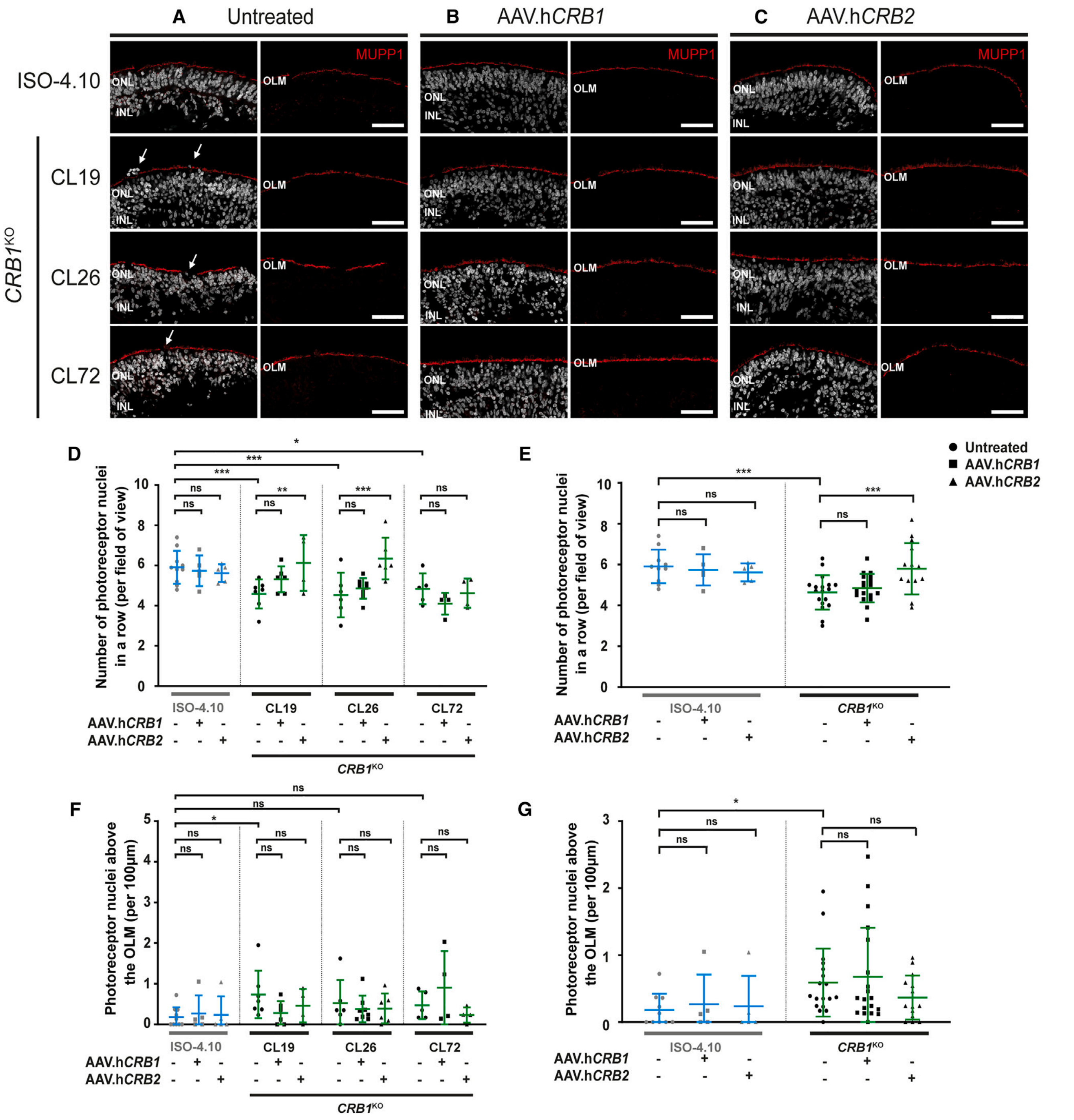

Figure 4. AAV-mediated gene therapy on *CRB1*^{KO} organoids transduced at DD120 shows an improved number of photoreceptor nuclei in a row

(A–C) Representative immunohistochemical images of (A) untreated, (B) AAV.h*CRB1*, or (C) AAV.h*CRB2* treated control and *CRB1*^{KO} retinal organoids at DD120 and analyzed at DD210. Stained with MUPP1 (red) at the OLM. (D–G) Quantification of the number of photoreceptor nuclei in a row (D and E) and above the OLM (F and G) per *CRB1*^{KO} clone (D and F) or all *CRB1*^{KO} clones combined (E and G). Each datapoint in the graph represents an individual organoid, of which an average has been taken of at least three representative images. The standard error of the mean (SEM) is derived from these averages. Number of individual organoids used for quantification per condition for untreated: 4.10 n = 10, *CRB1*^{KO} CL19 n = 7, CL26 n = 7, CL72 n = 5; AAV.h*CRB1* treated: 4.10 n = 5, *CRB1*^{KO} CL19 n = 6, CL26 n = 8, CL72 n = 4 from two independent differentiation batches; and AAV.h*CRB2* treated: 4.10 n = 5, *CRB1*^{KO} CL19 n = 4, CL26 n = 6, CL72 n = 3 from one differentiation batch. INL, inner nuclear layer; ONL, outer nuclear layer; OLM, outer limiting membrane. Scale bar, 50 μm, statistical analysis: generalized linear mixed models with $p < 0.05$ (*), $p < 0.01$ (**), and $p < 0.001$ (***).

and DD210 in *CRB1*^{KO} retinal organoids compared with the isogenic control. The data are similar to what was previously observed in *CRB1* RP patient-derived retinal organoids carrying missense mutations that allow the expression of a variant CRB1 protein.[26] Moreover, the complete loss of CRB1 in Müller glial cells of *Crb1* mouse retina also results in the protrusion of photoreceptor cell bodies into the subretinal space.[34–36] A more severe retinal phenotype in *Crb1*^{KO} mice was observed with concomitant loss of *Crb2* in Müller glial cells,[22,30,33,37] so this was investigated as well in the *CRB1*^{KO}*CRB2*$^{+/-}$ human retinal organoid model. A heterozygous mutation in *CRB2* targeting exon 3 was introduced in combination with a mutation targeting exon 2 of the *CRB1* gene, resulting in *CRB1*^{KO}*CRB2*$^{+/-}$ retinal organoids. These retinal organoids show a similar outer retina phenotype as the *CRB1*^{KO} and *CRB1* patient-derived retinal organoids.[25–27] Interestingly, the *CRB1*^{KO} and *CRB1*^{KO}*CRB2*$^{+/-}$ outer retina phenotypes studied here seem to be not much more severe than the outer retina phenotype previously observed in *CRB1* RP patient-derived retinal organoids.[25–27] The retinal organoids can show a variability in phenotype that can be caused by differences in genetic background, developmental age of the organoid, or practical handling differences between researchers during the organoid culture. To exclude differences in genetic background, the *CRB1*^{KO} and *CRB1*^{KO}*CRB2*$^{+/-}$ knockout mutations need to be introduced into the isogenic *CRB1* RP patient hiPSC. This will allow for a direct comparison between *CRB1* RP patient and *CRB1*^{KO} and *CRB1*^{KO}*CRB2*$^{+/-}$ retinal organoids with their isogenic controls all on the same genetic background. Furthermore, then the retinal organoids need to be cultured at the same time under the same culturing conditions in multiple batches of differentiation.

Interestingly, an inner and outer retinal phenotype was observed in *CRB1*^{KO} and *CRB1*^{KO}*CRB2*$^{+/-}$ retinal organoids compared with the isogenic control. This inner retinal phenotype was previously not observed in three independent *CRB1* RP patient-derived retinal organoids compared with their isogenic controls.[25–27] Staining for ISLET1/2-positive ON-cone and rod bipolar cells and SOX9-positive Müller glial cells showed abnormal localization of these cell types in the INL of *CRB1*^{KO} and *CRB1*^{KO}*CRB2*$^{+/-}$ retinal organoids. The data suggest that a complete lack of CRB1 in Müller glial cells and photoreceptors can result in a more advanced LCA-like phenotype affecting inner and outer retina. Interestingly, scRNA-seq analysis on DD230 *CRB1* patient-derived retinal organoids showed differences in gene expression profiles of Müller glial cells and photoreceptors, but changes were not detected in inner retinal cell types.[26] Future scRNA-seq studies on *CRB1*^{KO} and *CRB1*^{KO}*CRB2*$^{+/-}$ retinal organoids with their isogenic controls might provide insight in changes in the inner and outer retina. Moreover, the *CRB1* RP patient-derived retinal organoids had missense mutations and surprisingly showed a strong reduction in levels of variant CRB1 protein, whereas the *CRB1* mRNA transcript levels were not changed.[26] The *CRB1*^{KO} and *CRB1*^{KO}*CRB2*$^{+/-}$ retinal organoids showed complete loss of CRB1 leading to an inner and outer retina phenotype. scRNA-seq revealed that human Müller glial cells and photoreceptors express relatively high levels of *CRB1* transcripts, and that photoreceptors express relatively high levels of *CRB2*, while the Müller glial cells express relatively low levels of *CRB2*.[26] We hypothesize that relatively low levels of human *CRB2* in Müller glial cells are the cause for the phenotype in the inner retina of *CRB1*^{KO} retinal organoids. But, interestingly, mouse retinas that solely lack either CRB1 or CRB2 in Müller glial cells show very mild RP outer retinal phenotypes,[28,34–36] whereas a severe LCA-like retinal phenotype affecting inner and outer retina occurs in mouse retinas that lack both CRB1 and CRB2 in Müller glial cells.[24,37] Patients with missense variations in the *CRB1* gene can develop either early-onset RP or LCA or macular dystrophy.[16,17,19,20,38] Future studies need to show whether these differences in retinal phenotypes are due to relatively low levels of CRB2 and variant CRB1 in Müller glial cells.

All of the *CRB1*^{KO} and *CRB1*^{KO}*CRB2*$^{+/-}$ iPSC, but not the isogenic control iPSC from which the *CRB* mutant lines were derived, contained a CNV gain in chromosome 20q. This gain in chromosome 20q is one of the most common recurrent abnormalities in iPSCs and the biological significance of such recurrent abnormalities is still discussed.[32] Insights of CNV gains at 20q11.21 show that the differential gene expression pattern had a negative effect on the differentiation potential.[39] Genes associated with PI3K/AKT signaling pathway were significantly downregulated in the iPSC with a CNV gain at chromosome 20q, this pathway has an essential role in the survival of human pluripotent stem cells.[39] In our case, differentiating the *CRB1*^{KO} and *CRB1*^{KO}*CRB2*$^{+/-}$ hiPSC into retinal organoids was more challenging, a lower number of *CRB* knockout retinal organoids were produced, than for the isogenic control that gave relatively high numbers of retinal organoids. However, this could also be due to the quality of medium compounds (for example Matrigel), or other culture conditions, since we experienced variable production numbers of retinal organoids as well with other iPSC lines. Other groups also described the variability in the efficiency of differentiating certain iPSC lines efficiently into retinal organoids.[40] The potential effects of the CNV gain in chromosome 20q in the *CRB* knockout retinal organoids are unknown. The retinal phenotypes in the three *CRB1*^{KO} and three *CRB1*^{KO}*CRB2*$^{+/-}$ iPSC-derived organoids were similar or milder than the retinal phenotypes observed in three independent *CRB1* patient retinal organoids.[26] The CNV gain in chromosome 20q in the *CRB*^{KO} iPS cells did not result in an additional retinal phenotype. Negative effects on the differentiation potential of the *CRB*^{KO} and *CRB1*^{KO}*CRB2*$^{+/-}$ iPS cells cannot be excluded. Therefore, more research is needed if this CNV gain can have a negative effect on the differentiation potential into retinal organoids.

AAV tropism at two differentiation days was compared. Mainly photoreceptors and Müller glial cells were transduced on DD135 and DD200 retinal organoids with either AAV2.CMV.*GFP* or AAV5.CMV.*GFP*. Similar transduction was previously observed at DD120 and DD220.[25,26] Interestingly, in previous studies we observed efficient AAV5 transduction at DD220 of Müller glial cells in retinal organoids or in cadaver adult human retinal explants. Photoreceptors were not efficiently transduced with AAV5 in DD220 retinal organoids. Photoreceptors in cadaver adult human retinal

explants were only efficiently transduced with AAV5 in the presence of photoreceptor segments at the time of transduction.[25] In conclusion, we have shown that AAV2 and AAV5 are capable of infecting at DD120 and DD135 the Müller glial cells and photoreceptors in hiPSC-derived control retinal organoids.

Using AAV-mediated gene augmentation therapy, we have improved the outer retinal phenotype in *CRB1*^{KO} retinal organoids after AAV.h*CRB2* treatment. However, we did not observe a statistically significant improved number of photoreceptor nuclei in a row after AAV.h*CRB1* treatment of *CRB1*^{KO} retinal organoids. The AAV.h*CRB1* vector contains a minimal CMV promoter of 0.26 kb, whereas the AAV.h*CRB2* vector contains a full-length CMV promoter of 0.6 kb. Use of different promoters was needed to keep the size of the AAV-*CRB1* and AAV-*CRB2* vectors at 4.8 kb, which is close to the maximum packaging capacity of AAV. The short CMV promoter worked efficiently in mouse retina,[22,33,41] but the promoter activities might differ in human Müller glial cells and photoreceptors.[42] AAV-*CRB2* and AAV-*CRB1* transduction onto *CRB1* patient retinal organoids prevents morphological and transcriptional aberrations.[26,27] In these experiments, the number of protrusions of photoreceptor nuclei into the cell culture medium was effectively decreased for AAV.*hCRB2* but not for AAV.h*CRB1*. The less efficient morphological correction by AAV.h*CRB1* in *CRB1*^{KO} and *CRB1* patient retinal organoids could potentially be explained by the 4-fold lowered expression of a variant CRB1 protein in *CRB1* patient-derived retinal organoids and the complete absence of CRB1 protein in the *CRB1*^{KO} retinal organoids. We hypothesize that the *CRB1*^{KO} and *CRB1* patient retinal organoids might need higher levels of AAV.h*CRB1* in Müller glial cells to improve the outer retinal phenotype. Another possibility, as described above, could be because of modifying factors and differences in the genetic backgrounds of the *CRB1*^{KO} and *CRB1* patient retinal organoids. And of interest, the AAV.*CRB1* expression vector with the small 0.26-kb CMV promoter caused retinal toxicity upon subretinal or upon intravitreal injection in *Crb1* mice,[22,33,41] and in *in vitro* studies on *CRB1*^{KO} and *CRB1* patient retinal organoids the AAV.*CRB1* vector performs less efficient on preventing morphological aberrations than the AAV.*CRB2* vector in the current study and the study on *CRB1* patient retinal organoids.[26] *CRB1*^{KO} retinal organoids showed an improved retinal phenotype after AAV.h*CRB2* treatment. Using anti-CRB2 immunohistochemical staining, recombinant h*CRB2* protein was detected at the OLM.

In conclusion, we generated and differentiated *CRB1*^{KO} and *CRB1*^{KO}*CRB2*$^{+/-}$ hiPSC into retinal organoids and observed an extended phenotype compared with the phenotype observed in *CRB1*-patient-derived retinal organoids and in *Crb* mutant mouse studies. The *CRB1*^{KO} and *CRB1*^{KO}*CRB2*$^{+/-}$ retinal organoids showed an inner and outer retinal phenotype, whereas the *CRB1* patient-derived retinal organoids showed only an outer retinal phenotype. Using AAV-mediated gene augmentation therapy, we have improved the outer retinal phenotype in *CRB1*^{KO} retinal organoids. These data provide essential information for future gene therapy approaches for patients with mutations in the *CRB1* gene.

MATERIAL AND METHODS

Generation of *CRB1*^{KO} and *CRB1*^{KO}*CRB2*$^{+/-}$ hiPSC

Three *CRB1*^{KO} (CL19, CL26, and CL72) and three *CRB1*^{KO}*CRB2*$^{+/-}$ (CL4, CL9, and CL17) were generated from the hiPSC isogenic control (LUMC04iCTRL10) using CRISPR-Cas9 technology (Table S1) (Applied Stem Cell, California, USA). In short, two guide RNAs (gRNA) per gene of interest were designed, targeting exon 2 of *CRB1* and exon 3 of *CRB2*. These gRNAs were individually cloned into a gRNA/Cas9 expression vector by inserting double-stranded oligo cassettes of each gRNA between the two BbsI sites in the pBT-U6-Cas9-2A-GFP vector. Each oligo cassette consists of a 20-bp gRNA sequence with a guanosine at the 5′-end for optimal expression, and adherent ends for cloning at BbsI sites. Following construct delivery into the target cells, the abilities of these gRNAs to promote double-stranded breaks were evaluated using Sanger sequencing. gRNAs with the best targeting and repairing efficiency per gene of interest were selected for transfection of iPSC, gRNA for *CRB1* = GAAACTACCATTGGTT CCTG and for *CRB2* = AGAGCCAGCCGTGCGCACAT. After transfecting gRNA and Cas9 into the target cells, single-cell-derived clones were screened and three correct clones per gene of interest were selected and expanded for further use.

Confirmation of the mutation was done by PCR and subsequent Sanger sequencing for CRB1 and next generation sequencing (NGS) for CRB2 (Table S2).

NGS sample preparation and data analysis

Genomic DNA of hiPSC LUMC0004iCTRL10, and three *CRB1*^{KO}*CRB2*$^{+/-}$ (CL4, CL9, and CL17) were extracted using the DNeasy Blood & Tissue Kit protocol (Qiagen; 69506). The NGS first-round PCR for *CRB2* (Table S3), to amplify a target region with Illumina adapter overhang, was performed using a GoTaq G2 DNA Polymerase kit (Promega; M7845). After this, amplicons were purified by the AMPure XP kit (Beckman Coulter; A63881). Then the barcode PCR was performed to generate a library of amplicons using Illumina tag-specific primer pairs with unique sequence combinations for demultiplexing and sample identification (Table S3) using Kapa HiFi 2x Ready Mix (Roche; KK2602) and subsequently the purification using AMPure XP kit was carried out. The concentration was determined using Qubit2.0 fluorometer (Invitrogen) and Qubit dsDNA HS assay kit (Invitrogen; Q32854). The sample quality control was performed using capillarity electrophoresis with a 2100 BioAnalyzer (Agilent). The amplicons from each sample were pooled at equivalent DNA quantities. Finally, this library of pooled barcoded amplicons was subjected to Illumina MiSeq sequencing with 100k reads. The data were analyzed using CRISPResso2.[43,44]

Cell culture and retinal organoid differentiation

The following hiPSC lines were used for organoid differentiation: three *CRB1*^{KO} (CL19, CL26, and CL72) and three *CRB1*^{KO}*CRB2*$^{+/-}$ (CL4, CL9, and CL17) derived from the isogenic control (LUMC04iCTRL10) (Table S1). hiPSC lines were derived from skin fibroblasts using polycistronic Lentiviral vectors.[45] hiPSC were

cultured on Matrigel coated plates in mTeSR plus medium (STEMCELL Technologies) and passaged mechanically using gentle cell dissociation reagent. Retinal organoid differentiation was carried out as previously reported.[25–27,46,47] Variable numbers of rod and cone photoreceptors in different retinal organoids depending on culture conditions have been described.[47]

AAV transduction of hiPSC-derived retinal organoids

Two to three retinal organoids (about 4.10^5 cells per organoid) were plated in a single 96-well agarose-coated plate, multiple 96-wells were used per experiment, and were infected at DD120, DD135, or DD200 with the appropriate AAV concentration in 50 μL RLM2 medium.[46,48] Treated organoids were incubated at 5% CO_2 at 37°C, and 8 h later the organoids were topped up to 200 μL. The next day, the treated organoids were transferred to a 24-well plate and cultured until the desired differentiation day (for AAV.*CRB* treatment) or for three weeks (for AAV.*GFP* tropism). The following viral vectors were used: AAV2.CMV.*GFP* (105530-AAV2; Addgene), AAV5.CMV.*GFP* (105530-AAV5; Addgene), AAV5.CMVmin.h*CRB1* and AAV5.CMV.h*CRB2* (HORAMA) with a titer of $1x10^{10}$, $3.3x10^{10}$, $6.6x10^{10}$, or $10x10^{10}$ genome copies (gc) per well.

Immunohistochemical analysis

Organoids were collected and fixed with 4% paraformaldehyde in PBS for 20 min at room temperature (RT), then briefly washed in PBS and subsequently cryo-protected in 15% and 30% sucrose for at least 30 min. The samples were embedded in Tissue-Tek O.C.T. Compound (Sakura, Finetek) and stored at −20°C for future use. Then cryosections of 8 μM were made with a Leica CM1900 cryostat (Leica Microsystems). Slides with cryosections were stored at −20°C for future use.

For the immunohistochemical analysis, the slides were blocked in 10% normal goat serum, 0.4% Triton X-100, and 1% bovine serum albumin in PBS for 1 h at RT. Primary antibodies were incubated for least 3h at RT or overnight at 4°C with 0.3% normal goat serum, 0.4% Triton X-100, 1% BSA, and appropriate primary antibody concentration. Slides were washed twice in PBS, and incubated with a secondary antibody in 0.1% goat serum in PBS for 1 h at RT. Slides were then washed twice again in PBS, and mounted using Vectashield Hardset with DAPI mounting medium (H1800, Vector Laboratories, Burlingame, USA). The slides were imaged on a Leica TCS SP8 confocal microscope and images were processed using Leica Application Suite X (v3.7.0.20979).

The following primary antibodies were used: CRB1 AK2 (1:200; homemade = $CRB1^{INT}$, used for CRB1 if not otherwise specified), $CRB1^{EX}$ (1:200, Abnova H00023418-A01), CRB2 SK11 (1:200; homemade), PALS1 (1:200; homemade), rhodopsin (1:500; Millipore Cat# MAB5356), SOX9 (1:250; Millipore Cat# AB5535). ISLET1-2 (1:200, DSHB Developmental Studies Hybridoma Bank 39.4D5-c Islet-1 and Islet-2 homeobox), MUPP1 (1:200, BD Biosciences M98820), OTX2 (1; 200, Proteintech 13497-1-AP), and CRALBP (1:200, Abcam Ab15051).

The following secondary antibodies were used: goat anti-mouse, goat anti-rabbit, or goat anti-chicken IgGs conjugated to Alexa 488, Alexa 555, and Alexa 647 (1:1000, Abcam).

Quantification and statistical analysis

Images at 40× magnification were manually quantified using Fiji ImageJ (ImageJ 1.53f51). At least four organoids from at least two differentiation batches per condition with 3–6 representative images of each organoid were used for quantification. The exact number of organoids used per experiment is mentioned in the figure legends. In each image, three regions were quantified for the number of photoreceptor nuclei in a row, the number of protruding cells above the OLM, the retinal thickness, inner nuclear thickness, and ONL thickness. All measured datapoints were averaged per organoid and plotted in the graph; so that each dot is one organoid. Data were presented either per 100-μm retinal length or per field of view. Data presentation was performed using GraphPad Prism version 8 (GraphPad Software).

Statistical analysis was performed using IBM SPSS statistics (version 25). A generalized mixed model with treatment as a fixed effect was performed on all quantification parameters; all individual data points per image were used for statistical analysis. Each datapoint in the graph represents an individual organoid, of which an average has been taken of 3–6 representative images. The standard error of the mean (SEM) is derived from these averages. Significance is indicated in graphs as $p < 0.05$ (*), $p < 0.01$ (**), and $p < 0.001$ (***).

DATA AND CODE AVAILABILITY

Further information and requests for resources and reagents should be directed to and will be fulfilled by the corresponding author, Jan Wijnholds (j.wijnholds@lumc.nl).

SUPPLEMENTAL INFORMATION

Supplemental information can be found online at https://doi.org/10.1016/j.omtm.2023.101128.

ACKNOWLEDGMENTS

The authors would like to thank Chelsey Linnenbank for technical assistance, the LUMC light and electron microscope facility for help during image acquisition, and all the previous and current members of the Wijnholds lab for advice on experiments and the manuscript. This work was funded by The Netherlands Organization for Health Research and Development (ZonMw grant 43200004, to J.W.), research grant MDBR-19-131-CRB1 from the University of Pennsylvania Orphan Disease Center in partnership with the Curing Retinal Blindness Foundation (to J.W.), Bontius Stichting 32072-8231 (to J.W.), Leiden Regenerative Medicine Platform 2018-CRB1 (to J.W.), and HORAMA 41400-8231 (to J.W.).

AUTHOR CONTRIBUTIONS

Conceptualization, N.B. and J.W.; Formal analysis, N.B.; Investigation, N.B., X.L., C.A.A, M.O, and P.M.J.Q.; Data curation, N.B. and X.L.; Writing - original draft, N.B.; Writing - review & editing, all

authors; Visualization, N.B.; Supervision, J.W.; Project administration, J.W.; Funding acquisition, J.W.

DECLARATION OF INTERESTS

The authors declare no competing interests. The LUMC is the holder of patent number PCT/NL2014/050549, which describes the potential clinical use of CRB2. J.W. is listed as co-inventor of this patent and is an employee of the LUMC. Horama (currently known as Coave Therapeutics) partially sponsored this study.

REFERENCES

1. Tepass, U., Theres, C., and Knust, E. (1990). crumbs encodes an EGF-like protein expressed on apical membranes of Drosophila epithelial cells and required for organization of epithelia. Cell *61*, 787–799. https://doi.org/10.1016/0092-8674(90)90189-l.
2. den Hollander, A.I., Davis, J., van der Velde-Visser, S.D., Zonneveld, M.N., Pierrottet, C.O., Koenekoop, R.K., Kellner, U., van den Born, L.I., Heckenlively, J.R., Hoyng, C.B., et al. (2004). CRB1 mutation spectrum in inherited retinal dystrophies. Hum. Mutat. *24*, 355–369. https://doi.org/10.1002/humu.20093.
3. den Hollander, A.I., ten Brink, J.B., de Kok, Y.J., van Soest, S., van den Born, L.I., van Driel, M.A., van de Pol, D.J., Payne, A.M., Bhattacharya, S.S., Kellner, U., et al. (1999). Mutations in a human homologue of Drosophila crumbs cause retinitis pigmentosa (RP12). Nat. Genet. *23*, 217–221. https://doi.org/10.1038/13848.
4. Boon, N., Wijnholds, J., and Pellissier, L.P. (2020). Research Models and Gene Augmentation Therapy for CRB1 Retinal Dystrophies. Front. Neurosci. *14*, 860. https://doi.org/10.3389/fnins.2020.00860.
5. Quinn, P.M., Pellissier, L.P., and Wijnholds, J. (2017). The CRB1 Complex: Following the Trail of Crumbs to a Feasible Gene Therapy Strategy. Front. Neurosci. *11*, 175. https://doi.org/10.3389/fnins.2017.00175.
6. Ray, T.A., Cochran, K., Kozlowski, C., Wang, J., Alexander, G., Cady, M.A., Spencer, W.J., Ruzycki, P.A., Clark, B.S., Laeremans, A., et al. (2020). Comprehensive identification of mRNA isoforms reveals the diversity of neural cell-surface molecules with roles in retinal development and disease. Nat. Commun. *11*, 3328. https://doi.org/10.1038/s41467-020-17009-7.
7. Margolis, B. (2018). The Crumbs3 Polarity Protein. Cold Spring Harbor Perspect. Biol. *10*, a027961. https://doi.org/10.1101/cshperspect.a027961.
8. Roh, M.H., Makarova, O., Liu, C.J., Shin, K., Lee, S., Laurinec, S., Goyal, M., Wiggins, R., and Margolis, B. (2002). The Maguk protein, Pals1, functions as an adapter, linking mammalian homologues of Crumbs and Discs Lost. J. Cell Biol. *157*, 161–172. https://doi.org/10.1083/jcb.200109010.
9. Bulgakova, N.A., and Knust, E. (2009). The Crumbs complex: from epithelial-cell polarity to retinal degeneration. J. Cell Sci. *122*, 2587–2596. https://doi.org/10.1242/jcs.023648.
10. Bujakowska, K., Audo, I., Mohand-Saïd, S., Lancelot, M.E., Antonio, A., Germain, A., Léveillard, T., Letexier, M., Saraiva, J.P., Lonjou, C., et al. (2012). CRB1 mutations in inherited retinal dystrophies. Hum. Mutat. *33*, 306–315. https://doi.org/10.1002/humu.21653.
11. Corton, M., Tatu, S.D., Avila-Fernandez, A., Vallespín, E., Tapias, I., Cantalapiedra, D., Blanco-Kelly, F., Riveiro-Alvarez, R., Bernal, S., García-Sandoval, B., et al. (2013). High frequency of CRB1 mutations as cause of Early-Onset Retinal Dystrophies in the Spanish population. Orphanet J. Rare Dis. *8*, 20. https://doi.org/10.1186/1750-1172-8-20.
12. Vallespin, E., Cantalapiedra, D., Riveiro-Alvarez, R., Wilke, R., Aguirre-Lamban, J., Avila-Fernandez, A., Lopez-Martinez, M.A., Gimenez, A., Trujillo-Tiebas, M.J., Ramos, C., and Ayuso, C. (2007). Mutation screening of 299 Spanish families with retinal dystrophies by Leber congenital amaurosis genotyping microarray. Invest. Ophthalmol. Vis. Sci. *48*, 5653–5661. https://doi.org/10.1167/iovs.07-0007.
13. Verbakel, S.K., van Huet, R.A.C., Boon, C.J.F., den Hollander, A.I., Collin, R.W.J., Klaver, C.C.W., Hoyng, C.B., Roepman, R., and Klevering, B.J. (2018). Non-syndromic retinitis pigmentosa. Prog. Retin. Eye Res. *66*, 157–186. https://doi.org/10.1016/j.preteyeres.2018.03.005.
14. Motta, F.L., Salles, M.V., Costa, K.A., Filippelli-Silva, R., Martin, R.P., and Sallum, J.M.F. (2017). The correlation between CRB1 variants and the clinical severity of Brazilian patients with different inherited retinal dystrophy phenotypes. Sci. Rep. *7*, 8654. https://doi.org/10.1038/s41598-017-09035-1.
15. Zobor, D., Brühwiler, B., Zrenner, E., Weisschuh, N., and Kohl, S. (2023). Genetic and Clinical Profile of Retinopathies Due to Disease-Causing Variants in Leber Congenital Amaurosis (LCA)-Associated Genes in a Large German Cohort. Int. J. Mol. Sci. *24*, 8915. https://doi.org/10.3390/ijms24108915.
16. Daich Varela, M., Georgiou, M., Alswaiti, Y., Kabbani, J., Fujinami, K., Fujinami-Yokokawa, Y., Khoda, S., Mahroo, O.A., Robson, A.G., Webster, A.R., et al. (2023). CRB1-Associated Retinal Dystrophies: Genetics, Clinical Characteristics, and Natural History. Am. J. Ophthalmol. *246*, 107–121. https://doi.org/10.1016/j.ajo.2022.09.002.
17. Mairot, K., Smirnov, V., Bocquet, B., Labesse, G., Arndt, C., Defoort-Dhellemmes, S., Zanlonghi, X., Hamroun, D., Denis, D., Picot, M.C., et al. (2021). CRB1-Related Retinal Dystrophies in a Cohort of 50 Patients: A Reappraisal in the Light of Specific Muller Cell and Photoreceptor CRB1 Isoforms. Int. J. Mol. Sci. *22*, 12642. https://doi.org/10.3390/ijms222312642.
18. Nguyen, X.T.A., Talib, M., van Schooneveld, M.J., Wijnholds, J., van Genderen, M.M., Schalij-Delfos, N.E., Klaver, C.C.W., Talsma, H.E., Fiocco, M., Florijn, R.J., et al. (2022). CRB1-Associated Retinal Dystrophies: A Prospective Natural History Study in Anticipation of Future Clinical Trials. Am. J. Ophthalmol. *234*, 37–48. https://doi.org/10.1016/j.ajo.2021.07.021.
19. Karuntu, J.S., Nguyen, X.T.A., Talib, M., van Schooneveld, M.J., Wijnholds, J., van Genderen, M.M., Schalij-Delfos, N.E., Klaver, C.C.W., Meester-Smoor, M.A., van den Born, L.I., et al. (2023). Quality of life in patients with CRB1-associated retinal dystrophies: A longitudinal study. Acta Ophthalmol. https://doi.org/10.1111/aos.15769.
20. Talib, M., van Schooneveld, M.J., van Genderen, M.M., Wijnholds, J., Florijn, R.J., Ten Brink, J.B., Schalij-Delfos, N.E., Dagnelie, G., Cremers, F.P.M., Wolterbeek, R., et al. (2017). Genotypic and Phenotypic Characteristics of CRB1-Associated Retinal Dystrophies: A Long-Term Follow-up Study. Ophthalmology *124*, 884–895. https://doi.org/10.1016/j.ophtha.2017.01.047.
21. Talib, M., van Schooneveld, M.J., Wijnholds, J., van Genderen, M.M., Schalij-Delfos, N.E., Talsma, H.E., Florijn, R.J., Ten Brink, J.B., Cremers, F.P.M., Thiadens, A.A.H.J., et al. (2021). Defining inclusion criteria and endpoints for clinical trials: a prospective cross-sectional study in CRB1-associated retinal dystrophies. Acta Ophthalmol. *99*, e402–e414. https://doi.org/10.1111/aos.14597.
22. Buck, T.M., Vos, R.M., Alves, C.H., and Wijnholds, J. (2021). AAV-CRB2 protects against vision loss in an inducible CRB1 retinitis pigmentosa mouse model. Mol. Ther. Methods Clin. Dev. *20*, 423–441. https://doi.org/10.1016/j.omtm.2020.12.012.
23. van Rossum, A.G.S.H., Aartsen, W.M., Meuleman, J., Klooster, J., Malysheva, A., Versteeg, I., Arsanto, J.P., Le Bivic, A., and Wijnholds, J. (2006). Pals1/Mpp5 is required for correct localization of Crb1 at the subapical region in polarized Muller glia cells. Hum. Mol. Genet. *15*, 2659–2672. https://doi.org/10.1093/hmg/ddl194.
24. Quinn, P.M., Mulder, A.A., Henrique Alves, C., Desrosiers, M., de Vries, S.I., Klooster, J., Dalkara, D., Koster, A.J., Jost, C.R., and Wijnholds, J. (2019). Loss of CRB2 in Muller glial cells modifies a CRB1-associated retinitis pigmentosa phenotype into a Leber congenital amaurosis phenotype. Hum. Mol. Genet. *28*, 105–123. https://doi.org/10.1093/hmg/ddy337.
25. Quinn, P.M., Buck, T.M., Mulder, A.A., Ohonin, C., Alves, C.H., Vos, R.M., Bialecka, M., van Herwaarden, T., van Dijk, E.H.C., Talib, M., et al. (2019). Human iPSC-Derived Retinas Recapitulate the Fetal CRB1 CRB2 Complex Formation and Demonstrate that Photoreceptors and Muller Glia Are Targets of AAV5. Stem Cell Rep. *12*, 906–919. https://doi.org/10.1016/j.stemcr.2019.03.002.
26. Boon, N., Lu, X., Andriessen, C.A., Moustakas, I., Buck, T.M., Freund, C., Arendzen, C.H., Böhringer, S., Boon, C.J.F., Mei, H., and Wijnholds, J. (2023). AAV-mediated gene augmentation therapy of CRB1 patient-derived retinal organoids restores the histological and transcriptional retinal phenotype. Stem Cell Rep. *18*, 1123–1137. https://doi.org/10.1016/j.stemcr.2023.03.014.
27. Buck, T.M., Quinn, P.M.J., Pellissier, L.P., Mulder, A.A., Jongejan, A., Lu, X., Boon, N., Koot, D., Almushattat, H., Arendzen, C.H., et al. (2023). CRB1 is required for recycling by RAB11A+ vesicles in human retinal organoids. Stem Cell Rep. *18*, 1793–1810. https://doi.org/10.1016/j.stemcr.2023.07.001.

28. Alves, C.H., Pellissier, L.P., Vos, R.M., Garcia Garrido, M., Sothilingam, V., Seide, C., Beck, S.C., Klooster, J., Furukawa, T., Flannery, J.G., et al. (2014). Targeted ablation of Crb2 in photoreceptor cells induces retinitis pigmentosa. Hum. Mol. Genet. *23*, 3384–3401. https://doi.org/10.1093/hmg/ddu048.

29. Alves, C.H., Sanz, A.S., Park, B., Pellissier, L.P., Tanimoto, N., Beck, S.C., Huber, G., Murtaza, M., Richard, F., Sridevi Gurubaran, I., et al. (2013). Loss of CRB2 in the mouse retina mimics human retinitis pigmentosa due to mutations in the CRB1 gene. Hum. Mol. Genet. *22*, 35–50. https://doi.org/10.1093/hmg/dds398.

30. Pellissier, L.P., Lundvig, D.M.S., Tanimoto, N., Klooster, J., Vos, R.M., Richard, F., Sothilingam, V., Garcia Garrido, M., Le Bivic, A., Seeliger, M.W., and Wijnholds, J. (2014). CRB2 acts as a modifying factor of CRB1-related retinal dystrophies in mice. Hum. Mol. Genet. *23*, 3759–3771. https://doi.org/10.1093/hmg/ddu089.

31. Quinn, P.M., Alves, C.H., Klooster, J., and Wijnholds, J. (2018). CRB2 in immature photoreceptors determines the superior-inferior symmetry of the developing retina to maintain retinal structure and function. Hum. Mol. Genet. *27*, 3137–3153. https://doi.org/10.1093/hmg/ddy194.

32. Assou, S., Girault, N., Plinet, M., Bouckenheimer, J., Sansac, C., Combe, M., Mianné, J., Bourguignon, C., Fieldes, M., Ahmed, E., et al. (2020). Recurrent Genetic Abnormalities in Human Pluripotent Stem Cells: Definition and Routine Detection in Culture Supernatant by Targeted Droplet Digital PCR. Stem Cell Rep. *14*, 1–8. https://doi.org/10.1016/j.stemcr.2019.12.004.

33. Pellissier, L.P., Quinn, P.M., Alves, C.H., Vos, R.M., Klooster, J., Flannery, J.G., Heimel, J.A., and Wijnholds, J. (2015). Gene therapy into photoreceptors and Muller glial cells restores retinal structure and function in CRB1 retinitis pigmentosa mouse models. Hum. Mol. Genet. *24*, 3104–3118. https://doi.org/10.1093/hmg/ddv062.

34. van de Pavert, S.A., Kantardzhieva, A., Malysheva, A., Meuleman, J., Versteeg, I., Levelt, C., Klooster, J., Geiger, S., Seeliger, M.W., Rashbass, P., et al. (2004). Crumbs homologue 1 is required for maintenance of photoreceptor cell polarization and adhesion during light exposure. J. Cell Sci. *117*, 4169–4177. https://doi.org/10.1242/jcs.01301.

35. van de Pavert, S.A., Meuleman, J., Malysheva, A., Aartsen, W.M., Versteeg, I., Tonagel, F., Kamphuis, W., McCabe, C.J., Seeliger, M.W., and Wijnholds, J. (2007). A single amino acid substitution (Cys249Trp) in Crb1 causes retinal degeneration and deregulates expression of pituitary tumor transforming gene Pttg1. J. Neurosci. *27*, 564–573. https://doi.org/10.1523/JNEUROSCI.3496-06.2007.

36. van de Pavert, S.A., Sanz, A.S., Aartsen, W.M., Vos, R.M., Versteeg, I., Beck, S.C., Klooster, J., Seeliger, M.W., and Wijnholds, J. (2007). Crb1 is a determinant of retinal apical Muller glia cell features. Glia *55*, 1486–1497. https://doi.org/10.1002/glia.20561.

37. Pellissier, L.P., Alves, C.H., Quinn, P.M., Vos, R.M., Tanimoto, N., Lundvig, D.M.S., Dudok, J.J., Hooibrink, B., Richard, F., Beck, S.C., et al. (2013). Targeted ablation of CRB1 and CRB2 in retinal progenitor cells mimics Leber congenital amaurosis. PLoS Genet. *9*, e1003976. https://doi.org/10.1371/journal.pgen.1003976.

38. Tsang, S.H., Burke, T., Oll, M., Yzer, S., Lee, W., Xie, Y.A., and Allikmets, R. (2014). Whole exome sequencing identifies CRB1 defect in an unusual maculopathy phenotype. Ophthalmology *121*, 1773–1782. https://doi.org/10.1016/j.ophtha.2014.03.010.

39. Jo, H.Y., Lee, Y., Ahn, H., Han, H.J., Kwon, A., Kim, B.Y., Ha, H.Y., Kim, S.C., Kim, J.H., Kim, Y.O., et al. (2020). Functional in vivo and in vitro effects of 20q11.21 genetic aberrations on hPSC differentiation. Sci. Rep. *10*, 18582. https://doi.org/10.1038/s41598-020-75657-7.

40. Hallam, D., Hilgen, G., Dorgau, B., Zhu, L., Yu, M., Bojic, S., Hewitt, P., Schmitt, M., Uteng, M., Kustermann, S., et al. (2018). Human-Induced Pluripotent Stem Cells Generate Light Responsive Retinal Organoids with Variable and Nutrient-Dependent Efficiency. Stem Cell. *36*, 1535–1551. https://doi.org/10.1002/stem.2883.

41. Pellissier, L.P., Hoek, R.M., Vos, R.M., Aartsen, W.M., Klimczak, R.R., Hoyng, S.A., Flannery, J.G., and Wijnholds, J. (2014). Specific tools for targeting and expression in Muller glial cells. Mol. Ther. Methods Clin. Dev. *1*, 14009. https://doi.org/10.1038/mtm.2014.9.

42. Buck, T.M., and Wijnholds, J. (2020). Recombinant Adeno-Associated Viral Vectors (rAAV)-Vector Elements in Ocular Gene Therapy Clinical Trials and Transgene Expression and Bioactivity Assays. Int. J. Mol. Sci. *21*, 4197. https://doi.org/10.3390/ijms21124197.

43. Clement, K., Rees, H., Canver, M.C., Gehrke, J.M., Farouni, R., Hsu, J.Y., Cole, M.A., Liu, D.R., Joung, J.K., Bauer, D.E., and Pinello, L. (2019). CRISPResso2 provides accurate and rapid genome editing sequence analysis. Nat. Biotechnol. *37*, 224–226. https://doi.org/10.1038/s41587-019-0032-3.

44. Wang, Q., Liu, J., Janssen, J.M., Le Bouteiller, M., Frock, R.L., and Gonçalves, M.A.F.V. (2021). Precise and broad scope genome editing based on high-specificity Cas9 nickases. Nucleic Acids Res. *49*, 1173–1198. https://doi.org/10.1093/nar/gkaa1236.

45. Warlich, E., Kuehle, J., Cantz, T., Brugman, M.H., Maetzig, T., Galla, M., Filipczyk, A.A., Halle, S., Klump, H., Schöler, H.R., et al. (2011). Lentiviral vector design and imaging approaches to visualize the early stages of cellular reprogramming. Mol. Ther. *19*, 782–789. https://doi.org/10.1038/mt.2010.314.

46. Quinn, P.M., Buck, T.M., Ohonin, C., Mikkers, H.M.M., and Wijnholds, J. (2018). Production of iPS-Derived Human Retinal Organoids for Use in Transgene Expression Assays. Methods Mol. Biol. *1715*, 261–273. https://doi.org/10.1007/978-1-4939-7522-8_19.

47. Zhong, X., Gutierrez, C., Xue, T., Hampton, C., Vergara, M.N., Cao, L.H., Peters, A., Park, T.S., Zambidis, E.T., Meyer, J.S., et al. (2014). Generation of three-dimensional retinal tissue with functional photoreceptors from human iPSCs. Nat. Commun. *5*, 4047. https://doi.org/10.1038/ncomms5047.

48. Buck, T.M., Pellissier, L.P., Vos, R.M., van Dijk, E.H.C., Boon, C.J.F., and Wijnholds, J. (2018). AAV Serotype Testing on Cultured Human Donor Retinal Explants. Methods Mol. Biol. *1715*, 275–288. https://doi.org/10.1007/978-1-4939-7522-8_20.

OMTM, Volume *31*

Supplemental information

Characterization and AAV-mediated *CRB* gene augmentation in human-derived *CRB1*^{KO} and *CRB1*^{KO}*CRB2*$^{+/-}$ retinal organoids

Nanda Boon, Xuefei Lu, Charlotte A. Andriessen, Michaela Orlovà, Peter M.J. Quinn, Camiel J.F. Boon, and Jan Wijnholds

Table S1. hiPSC line information, related to Figure S1.

Previously published	Description	Gender
LUMC04iCTRL10	ISO-4.10 = Isogenic control	Male
No	*CRB1*^{KO} CL19 = homozygous c.500del ; p.(Ser44Serfs*)	Male
No	*CRB1*^{KO} CL26 = homozygous c.500del ; p.(Ser44Serfs*)	Male
No	*CRB1*^{KO} CL72 = homozygous c.500del ; p.(Ser44Serfs*)	Male
No	*CRB1*^{KO}*CRB2*$^{+/-}$ CL4 =<br>*CRB1*: homozygous c.500del ; p.(Ser44Serfs*),<br>*CRB2*: heterozygous c.576_598del; p.(Cys193Argfs*)	Male
No	*CRB1*^{KO}*CRB2*$^{+/-}$ CL9 =<br>*CRB1*: homozygous c.500del ; p.(Ser44Serfs*),<br>*CRB2*: heterozygous c.576_598del; p.(Cys193Argfs*)	Male
No	*CRB1*^{KO}*CRB2*$^{+/-}$ CL17 =<br>*CRB1*: homozygous c.498_507delinsTGCC ; p.(Ser44Lysfs*),<br>*CRB2*: heterozygous c.583_584del; p.(His195Trpfs*)	Male

Table S2. PCR primer sequences for confirmation mutations.

Target		PCR primers
CRB1	FW	GACAATGATTGTTCTTGTTCAGACACAGCC
	REV	CATCCACTTCCAAGTCGCAGTGTC
		**Sanger sequencing primers**
CRB1	FW	GGACAAAGACTGTGACAACATGAAAGACC
	REV	GGACACAGAAGCAGGAGTAACCATC
**Target**		**NGS primers (5' to 3') (Illumina adapter overhang in blue)**
CRB2	FW	GATGTGTATAAGAGACAGGTGTCCATCCTGCACCCTGTG
	REV	CGTGTGCTCTTCCGATCTTCGCTCACCCGTTGACCAGGT

Table S3. Barcode PCR primers used in the NGS amplicon sequence analysis.

Barcode name	Primer Sequence (5' to 3')	Barcode in primer	Read in Miseq
719	CAAGCAGAAGACGGCATACGAGAT | TACTACGC | GTGACTGGAGTTCAGACGTGTGCTCTTCCGATCT	TACTACGC	TACTACGC
720	CAAGCAGAAGACGGCATACGAGAT | AGGCTCCG | GTGACTGGAGTTCAGACGTGTGCTCTTCCGATCT	AGGCTCCG	AGGCTCCG
721	CAAGCAGAAGACGGCATACGAGAT | GCAGCGTA | GTGACTGGAGTTCAGACGTGTGCTCTTCCGATCT	GCAGCGTA	GCAGCGTA
722	CAAGCAGAAGACGGCATACGAGAT | GAGCGCTA | GTGACTGGAGTTCAGACGTGTGCTCTTCCGATCT	GAGCGCTA	GAGCGCTA
508	AATGATACGGCGACCACCGAGATCTACAC | GTACTGAC | TCGTCGGCAGCGTCAGATGTGTATAAGAGACAG	GTACTGAC	GTACTGAC

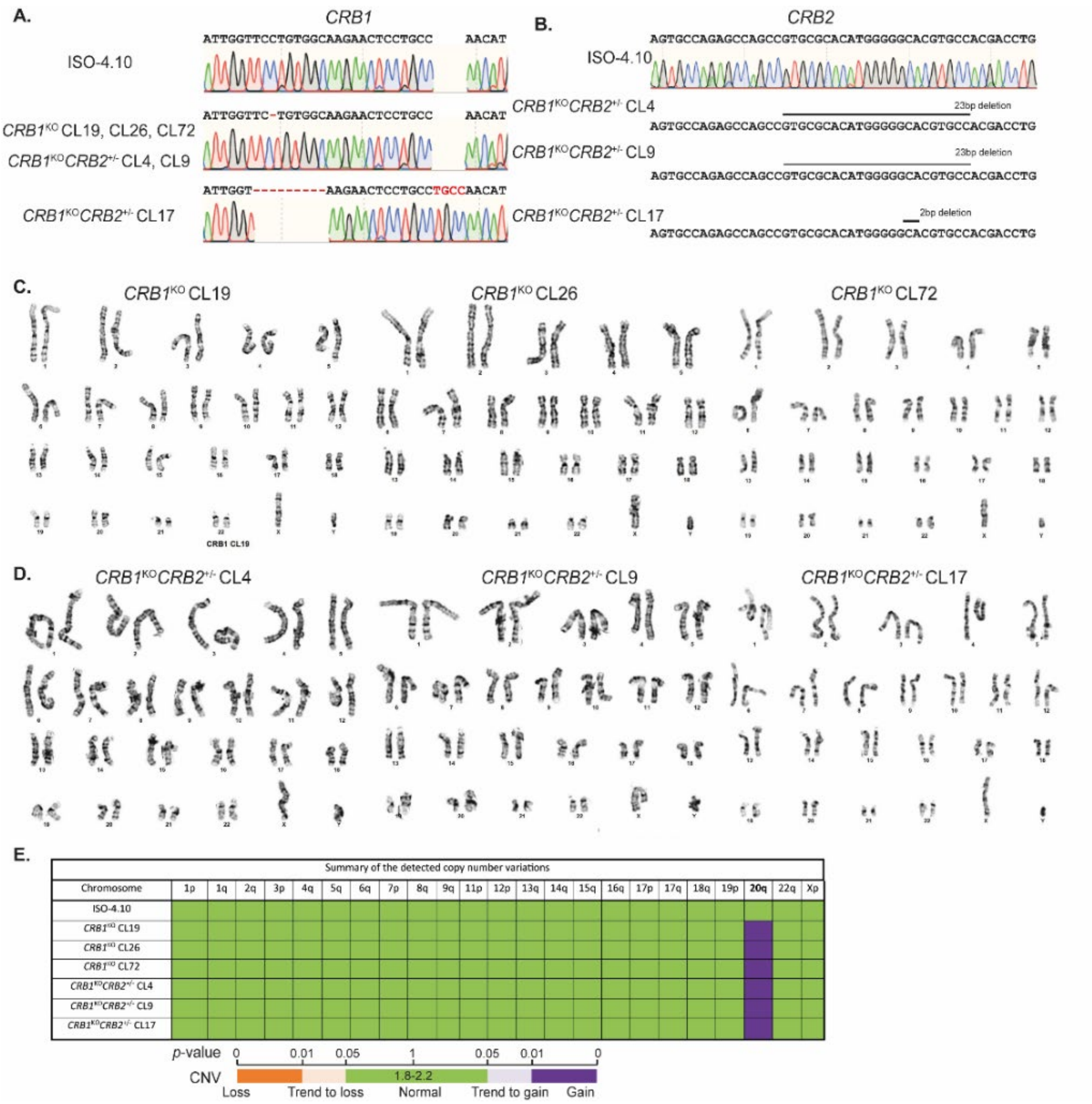

Figure S1: Confirmation of mutations and condition of *CRB1*^{KO} and *CRB1*^{KO}*CRB2*$^{+/-}$ hiPSC. Related to all figures. (A) Confirmation of the homozygous *CRB1* mutation in *CRB1*^{KO} and *CRB1*^{KO}*CRB2*$^{+/-}$ hiPSC using Sanger sequencing, with *CRB1*^{KO}*CRB2*$^{+/-}$ CL17 containing a distinct mutation. (B) Confirmation of the heterozygous *CRB2* mutation in *CRB1*^{KO}*CRB2*$^{+/-}$ hiPSC using Sanger sequencing (upper trace) and an insertion/deletion (INDEL) detection software (ICE) (lower sequences). (C, D) Karyotyping results of (C) *CRB1*^{KO} and (D) *CRB1*^{KO}*CRB2*$^{+/-}$ hiPSC. (E) Meta-analysis of 90% most recurrent abnormalities in hiPSCs showing a gain in copy number variation (CNV) in chromosome 20q for hiPSC used in this study.

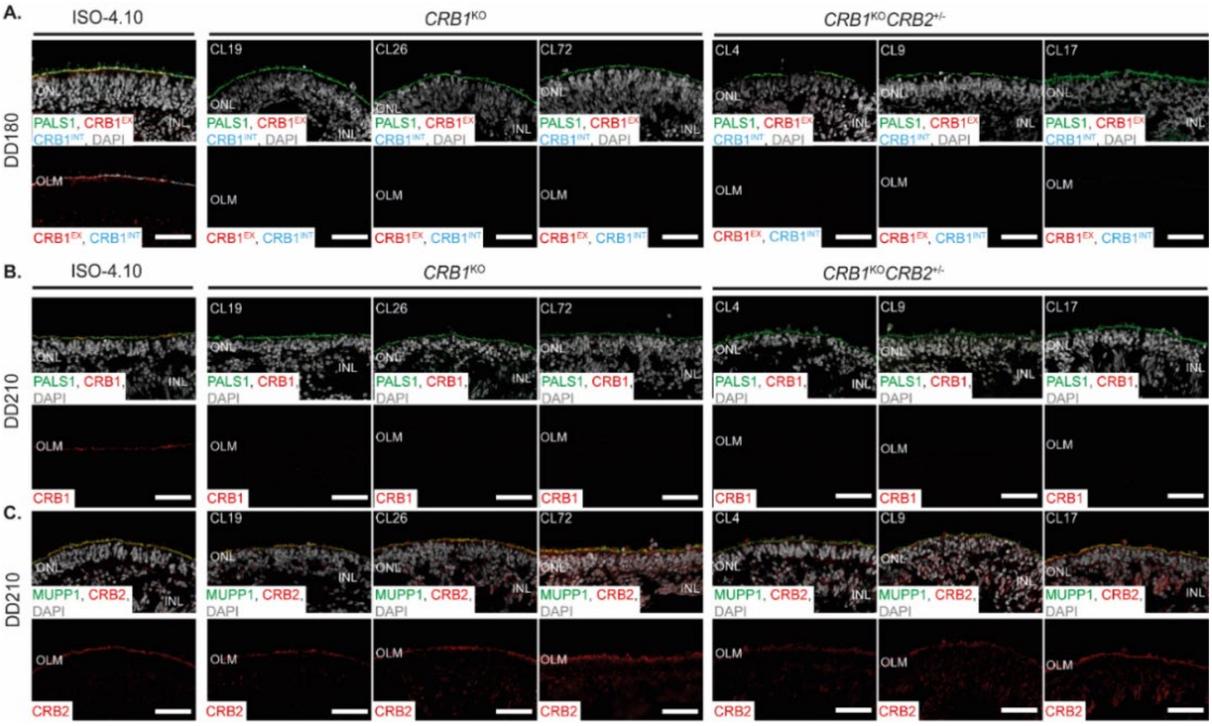

Figure S2: CRB1 is absent in DD180 and DD210 *CRB1*^{KO} and *CRB1*^{KO}*CRB2*$^{+/-}$ retinal organoids. Related to Figure 2. Representative immunohistochemical images of three *CRB1*^{KO} and three *CRB1*^{KO}*CRB2*$^{+/-}$ retinal organoids compared to the isogenic control at DD180 and DD210. (A) DD180 organoids expressing PALS1 (green) in all retinal organoids at the OLM and CRB1^{EX} (red), and CRB1^{INT} (cyan) was only detected in the isogenic control. (B) DD210 retinal organoids stained with PALS1 (green) and CRB1 (red), similar as observed in DD180. (C) MUPP1 (green) and CRB2 (red) is expressed at the OLM in control, *CRB1*^{KO}, and *CRB1*^{KO}*CRB2*$^{+/-}$ retinal organoids at DD210. Note: INL = Inner Nuclear Layer, ONL = Outer Nuclear Layer, OLM = Outer Limiting Membrane. Scalebar = 50µm.

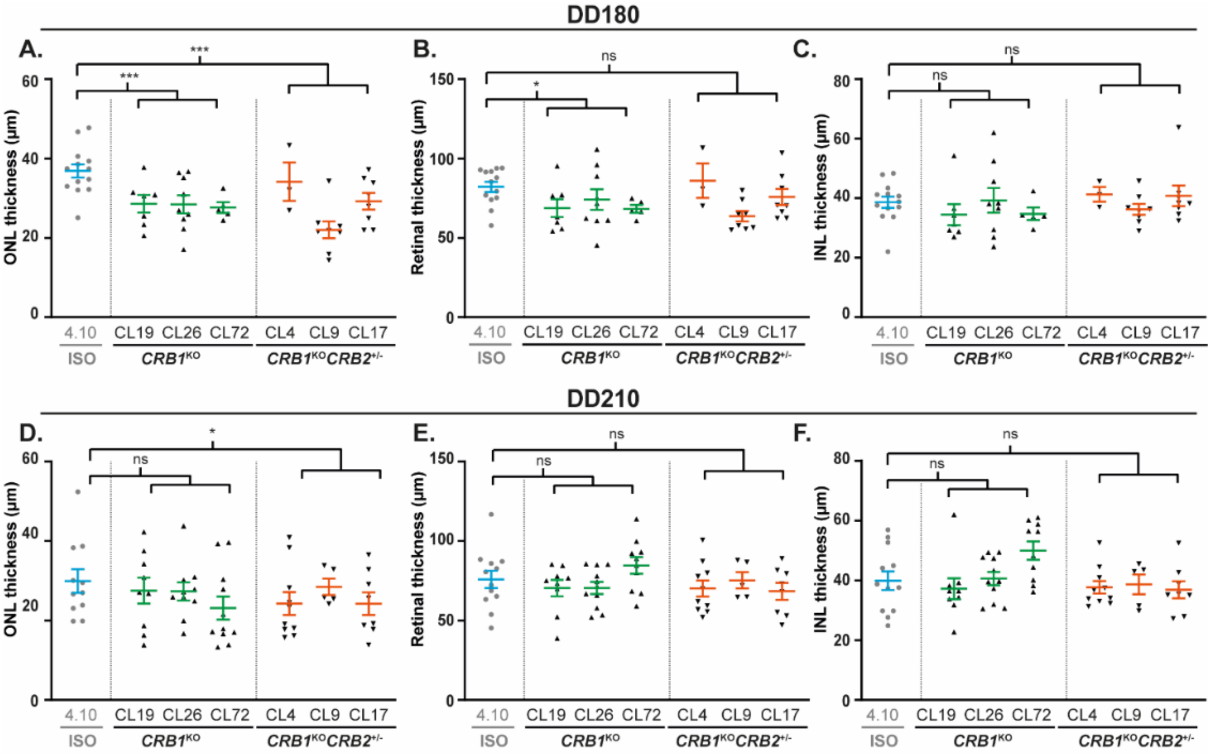

Figure S3: Additional phenotype quantifications of DD180 and DD210 *CRB1*^{KO} and *CRB1*^{KO}*CRB2*$^{+/-}$ retinal organoids. (A, B, C) Quantification of the (A) ONL, (B) retinal, and (C) INL thickness of DD180 retinal organoids. (D, E, F) Quantification of the (D) ONL, (E) retinal, and (F) INL thickness of DD210 retinal organoids. Each datapoint in the graph represents an individual organoid, of which an average has been taken of 3-6 representative images. The standard error of mean (SEM) is derived from these averages. Number of individual organoids used for the quantification per condition at DD180: 4.10 *n* =14, CRB1^{KO} CL19 *n* = 7, CL26 *n* =5, CL72 *n* = 9, *CRB1*^{KO}*CRB2*$^{+/-}$ CL4 *n* = 3, CL9 *n* = 8, CL17 *n* = 8; and DD210: 4.10 *n* = 12, CRB1^{KO} CL19 *n* = 9, CL26 *n* =10, CL72 *n* = 11, *CRB1*^{KO}*CRB2*$^{+/-}$ CL4 *n* = 8, CL17 *n* = 8 from at least two independent differentiation batches and *CRB1*^{KO}*CRB2*$^{+/-}$ CL9 *n* = 5 from one differentiation batch. Statical analysis: generalized linear mixed models with p<0.05 (*), p<0.01 (**), and p<0.001 (***).

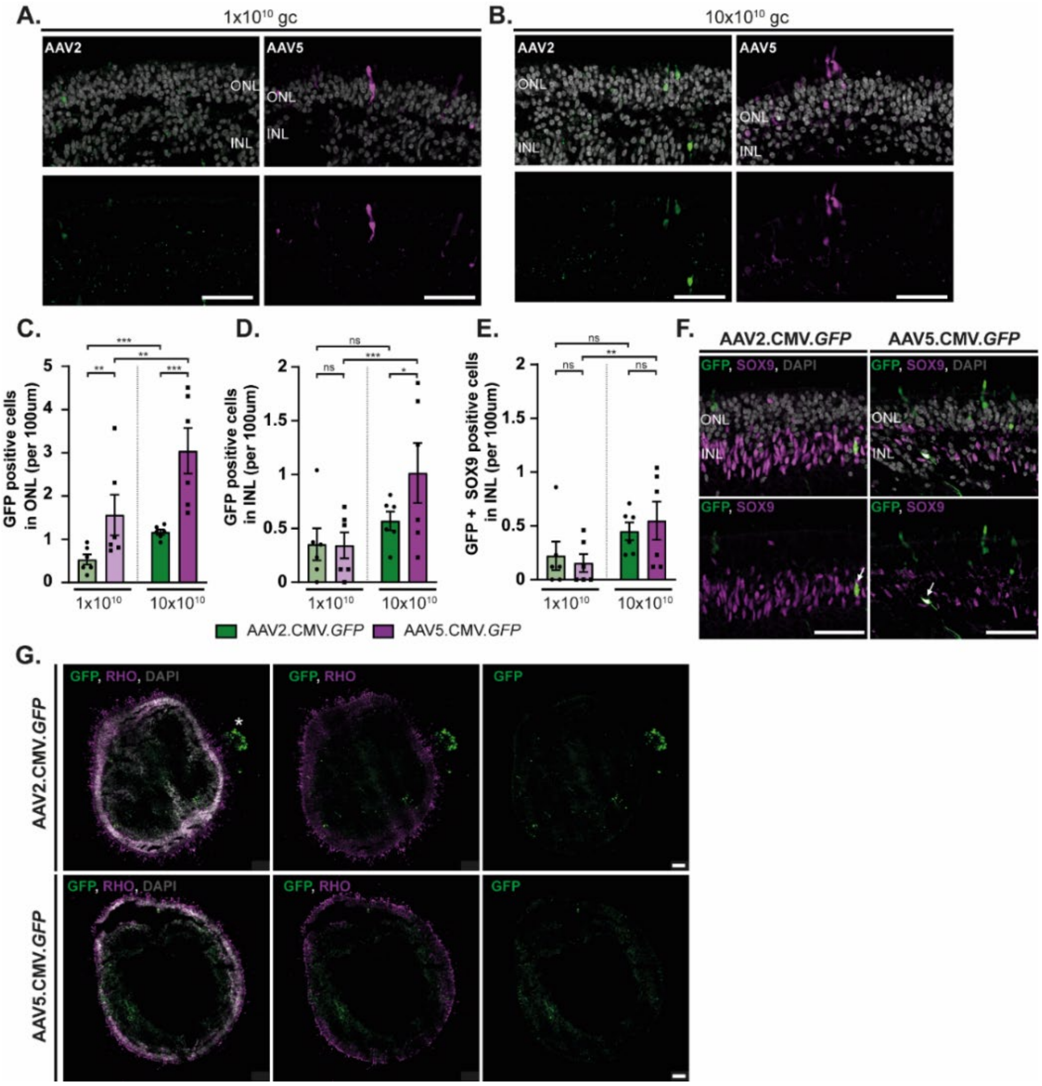

Figure S4: AAV transduction study of control retinal organoids transduced at DD200 with AAV2.CMV.*GFP* or AAV5.CMV.*GFP* (A, B) Representative immunohistochemical images of control retinal organoids transduced with (A) 1x10^{10}gc, or (B) 10x10^{10}gc AAV2.CMV.*GFP* or AAV5.CMV.*GFP*. (C,D, E) Quantification of the number of GFP positive cells in the (D) ONL, (E) INL, and (F) GFP positive and co-localized with SOX9 in the INL (marking Müller glial cells). Statistical analysis was performed within the same AAV capsid (dose-dependent) or within the same AAV titer (comparing AAV2 with AAV5 transduction efficiency). (F, G) Immunohistochemical images of co-localization of AAV.*GFP* with Müller glial cells marker SOX9 (F) and photoreceptor cell marker Rhodopsin (RHO) (G). Arrows indicate colocalization of SOX9 with GFP (F), and the asterisk indicates the RPE (G). Each datapoint in the graph represents individual organoids, of which an average has been taken of 3-6 representative images. The standard error of mean (SEM) is derived from these averages. Number of individual organoids used for quantification per condition for AAV2.CMV.*GFP*: 1x10^{10}gc n = 6, 10x10^{10}gc n = 6; and for AAV5.CMV.*GFP*: 1x10^{10}gc n = 6, 10x10^{10}gc n = 6 from one differentiation batch. Note: INL = Inner Nuclear Layer, ONL = Outer Nuclear Layer. Scalebar = 50µm, statical analysis: generalized linear mixed models with p<0.05 (*), p<0.01 (**), and p<0.001 (***).

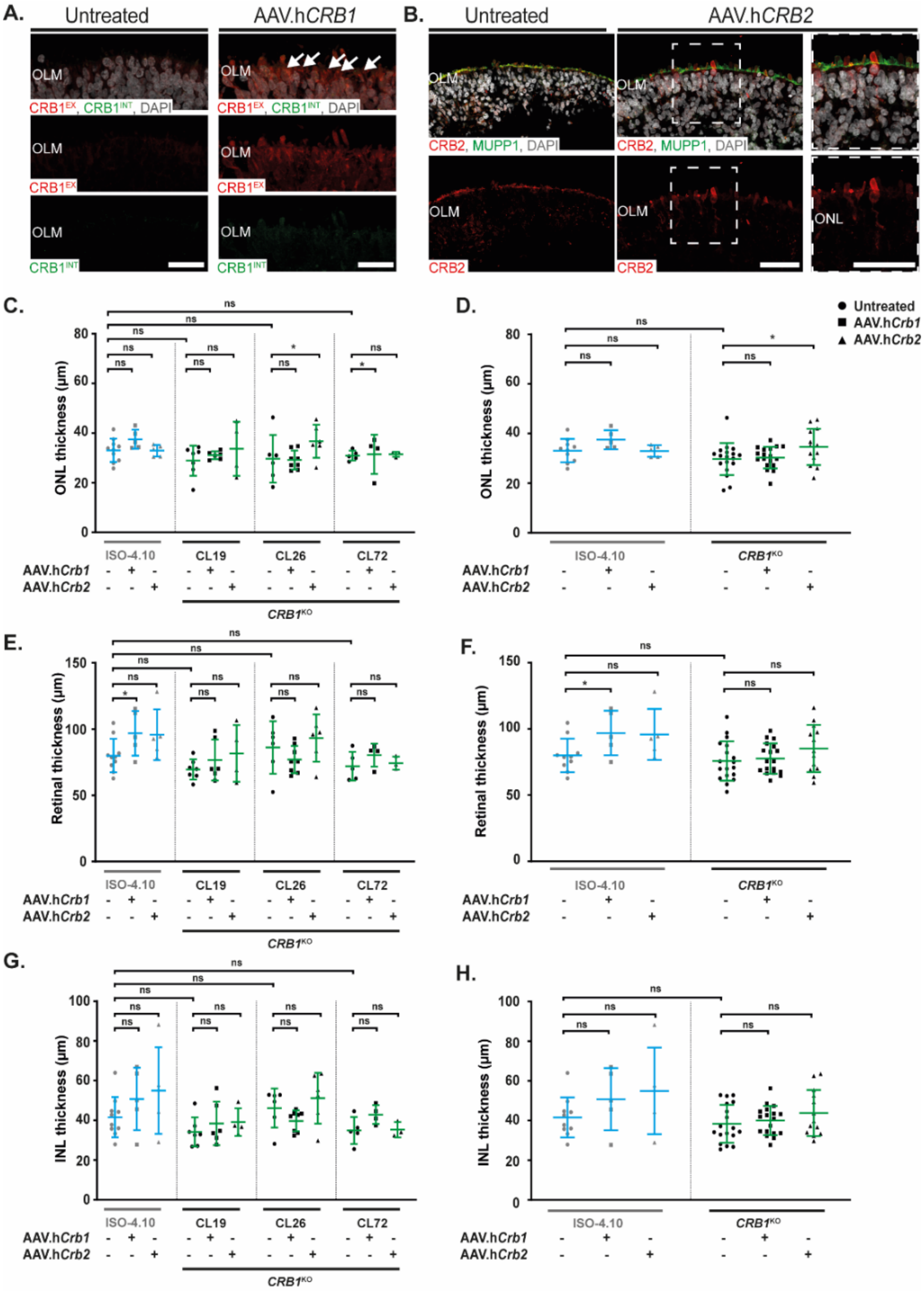

Figure S5: AAV-mediated gene therapy on *CRB1*^{KO} organoids. (A, B) representative immunohistochemical image of (A) CRB1 in untreated and AAV.h*CRB1* and (B) CRB2 in untreated and AAV.h*CRB2* treated *CRB1*^{KO} retinal organoids. (C,D, E, F, G, H) Quantification of the (C, D) ONL thickness, (E, F) the retinal thickness, and (G, H) INL thickness per *CRB1*^{KO} clone (C, E, G) or all *CRB1*^{KO} clones combined (D, F, H). Each datapoint in the graph represents individual organoids, of which an average has been taken of at least three representative images. The standard error of mean (SEM) is derived from these averages. Number of individual organoids used for quantification per condition for untreated: 4.10 n = 10, *CRB1*^{KO} CL19 n = 7, CL26 n = 7, CL72 n = 5; AAV.h*CRB1* treated: 4.10 n = 5, *CRB1*^{KO} CL19 n = 6, CL26 n = 8, CL72 n = 4 from two independent differentiation batches; and AAV.h*CRB2* treated: 4.10 n = 5, *CRB1*^{KO} CL19 n = 4, CL26 n = 6, CL72 n = 3 from one differentiation batch. Note: ONL = Outer Nuclear Layer, OLM = Outer Limiting Membrane. Scalebar = 50µm, statistical analysis: generalized linear mixed models with $p < 0.05$ (*), $p < 0.01$ (**), and $p < 0.001$ (***).
